# The Specificity and Broad Multitarget Properties of Ligands for the Free Fatty Acid Receptors FFA3/GPR41 and FFA2/GPR43 and the Related Hydroxycarboxylic Acid Receptor HCA2/GPR109A

**DOI:** 10.3390/ph14100987

**Published:** 2021-09-28

**Authors:** Egils Bisenieks, Brigita Vigante, Ramona Petrovska, Baiba Turovska, Ruslan Muhamadejev, Vitalijs Soloduns, Astrida Velena, Karlis Pajuste, Luciano Saso, Janis Klovins, Gunars Duburs, Ilona Mandrika

**Affiliations:** 1 Latvian Institute of Organic Synthesis, LV-1006 Riga, Latvia; egils.bisenieks@osi.lv (E.B.); vigante@osi.lv (B.V.); turovska@osi.lv (B.T.); muhamadejev@osi.lv (R.M.); vs002222@gmail.com (V.S.); astrida@osi.lv (A.V.); kpajuste@osi.lv (K.P.); 2 Latvian Biomedical Research and Study Centre, LV-1067 Riga, Latvia; ramona@biomed.lu.lv (R.P.); klovins@biomed.lu.lv (J.K.); 3 Department of Physiology and Pharmacology “Vittorio Erspamer”, University Sapienza, 00185 Rome, Italy; luciano.saso@uniroma1.it

**Keywords:** G-protein-coupled receptors, multi-target ligands, FFA3/GPR41, FFA2/GPR43, HCA2/GPR109A, electron-donating compounds

## Abstract

The paradigm of ligand-receptor interactions postulated as “one compound—one target” has been evolving; a multi-target, pleiotropic approach is now considered to be realistic. Novel series of 1,4,5,6,7,8-hexahydro-5-oxoquinolines, pyranopyrimidines and S-alkyl derivatives of pyranopyrimidines have been synthesized in order to characterise their pleiotropic, multitarget activity on the FFA3/GPR41, FFA2/GPR43, and HCA2/GPR109A receptors. Hexahydroquinoline derivatives have been known to exhibit characteristic activity as FFA3/GPR41 ligands, but during this study we observed their impact on FFA2/GPR43 and HCA2/GPR109A receptors as well as their electron-donating activity. Oxopyranopyrimidine and thioxopyranopyrimidine type compounds have been studied as ligands of the HCA2/GPR109A receptor; nevertheless, they exhibited equal or higher activity towards FFA3/GPR41 and FFA2/GPR43 receptors. S-Alkyl derivatives of pyranopyrimidines that have not yet been studied as ligands of GPCRs were more active towards HCA2/GPR109A and FFA3/GPR41 receptors than towards FFA2/GPR43. Representative compounds from each synthesized series were able to decrease the lipopolysaccharide-induced gene expression and secretion of proinflammatory cytokines (IL-6, TNF-α) and of a chemokine (MCP-1) in THP-1 macrophages, resembling the effect of HCA2/GPR109A ligand niacin and the endogenous ligand propionate. This study revealed groups of compounds possessing multitarget activity towards several receptors. The obtained data could be useful for further development of multitarget ligands.

## 1. Introduction

The paradigm of ligand-receptor interactions postulated as “one compound—one target” has been transformed over the years by the recognition that a multi-target, pleiotropic approach can also be remarkably productive or even preferable [1].

G-protein-coupled receptors (GPCRs) provide attractive targets for investigating the putative pleiotropy of ligands. GPCRs, with more than 800 members, constitute the largest family of membrane proteins that are widely expressed in the human body and are involved in the regulation of most physiological functions through the modulation of cell signaling pathways via G-proteins or β-arrestin. Consequently, GPCRs are important targets of many synthetic ligands. At least 108 GPCRs serve as targets for 475 (34%) of the drugs approved by United States Food and Drug Administration, and 68 potentially novel receptors are targeted by 320 (20%) of the drug candidates that are in clinical trials [2]. At the same time, the overwhelming majority of GPCRs (~220) still have not been successfully targeted by such small molecules as ligands.

The free fatty acid receptors 2 and 3 (FFA2/GPR43 and FFA3/GPR41) and the hydroxycarboxylic acid receptor 2 (HCA2/GPR109A) belong to the class of GPCRs that are activated by the products of dietary fiber digestion: metabolic intermediates such as short-chain fatty acids (acetate, propionate, butyrate) and hydroxylated carboxylic acids [3,4].

The FFA and HCA receptors are coupled to the pertussis toxin-sensitive, G_i/o_-alpha family of G- proteins, and FFA2 is also coupled to the G_q_ family. Their activation results in the inhibition of intracellular cAMP accumulation and mobilisation of intracellular calcium, respectively. These receptors are coexpressed in intestinal epithelial cells, pancreatic β-cells, adipocytes, and immune cells such as neutrophils, monocytes, and intestinal Treg cells, and exhibit some overlap in agonist (butyrate) specificity [5,6]. They are involved in multiple regulatory pathways affecting several important biological processes, such as gastrointestinal and immune functions and the regulation of energy homeostasis.

The FFA2/GPR43 and FFA3/GPR41 receptors are involved in energy homeostasis via the modulation of hormones, such as glucagon-like peptide 1, gastric inhibitory peptide, and peptide YY, affecting secretion from intestinal endocrine cells, suppression of fat accumulation in adipose tissue, and leading to increased insulin sensitivity [5,7]. The FFA3/GPR41 receptor also enhances insulin sensitivity through the gut-brain neural circuit, involving the activation of peripheral nerves [8]. The protective effects of propionate on the blood-brain barrier have been shown to occur by binding to FFA3/GPR41 on the surface of human brain endothelial cells, leading to a reduction in inflammatory and oxidative processes [9]. The FFA2/GPR43, FFA3/GPR41, and HCA2/GPR109A ligands reduce colonic inflammation by regulating the production of cytokines and chemokines by immune system cells. This mechanism has been shown to affect inflammatory responses in models of asthma, colitis, and arthritis [10,11,12].

Therefore, FFARs are regarded as novel drug targets for the treatment of metabolic disorders such as obesity, type 2 diabetes, gastrointestinal disorders, colorectal cancer, and diseases of the immune system [13,14,15]. 

There have been numerous attempts to approach the problem of obtaining selective and effective ligands for modulating biochemical pathways that involve metabolite GPCRs [2,16,17,18,19,20,21,22]. According to our knowledge, there has been no comparative study of the activities of putative ligands on these three GPCRs, namely, the free fatty acid receptors FFA2/GPR43 and FFA3/GPR41 and the hydroxycarboxylic acid receptor HCA2/GPR109A. A multitarget, pleiotropic approach can in principle enable the discovery of pharmaceutical agents combining the therapeutic effects of two or three separate drugs. Such an achievement could reduce the range of side effects, lower the costs of drug development and manufacturing, and possibly shorten drug development times. Already, 33% (156) of approved drugs targeting GPCRs have more than one therapeutic indication [23,24].

Rationally designed polypharmacological studies using a set of ligands or multi-target directed ligands have been proposed as the only approach that can confront the complexity of multifactorial diseases, e.g., Alzheimer’s disease [25,26,27,28,29].

The aim of this study was to determine the selectivity and multitarget characteristics of synthetic ligands for free fatty acid receptors (such as FFA2/GPR43 and FFA3/GPR41) and hydroxycarboxylic acid receptors. 

It would be useful to test the activity of hydroxycarboxylic acid receptor ligands on the free fatty acid receptors FFA3/GPR41 and FFA2/GPR43 and, vice versa, the activity of short-chain fatty acid receptor ligands on the hydroxycarboxylic acid receptor HCA2/GPR109A. As a result of this cross-examination, common structural patterns of hydroxycarboxylic acid receptor ligands and short-chain fatty acid receptor ligands may be revealed, leading to a broader scope of applications for such compounds.

**Figure 1:**
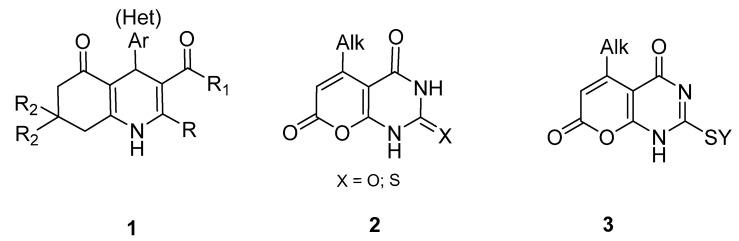
General structures of studied groups of compounds.

Therefore, we synthesized and studied three groups of compounds (Figure 1): 5-oxo-4-(aryl, heteryl)-1,4,5,6,7,8-hexahydroquinolines (1,4,5,6,7,8-hexahydro-5-oxoquinolines, HHQ)**,** (**1**) as known ligands of the FFA3/GPR41 and FFA2/GPR43 receptors, in order to characterise their impact on the HCA2/GPR109A receptor; (**2**) pyranopyrimidinetriones and thioxopyranopyrimidinediones (pyranopyrimidines), as known ligands of the HCA2/GPR109A receptor, in order to characterise their activity on the FFA3/GPR41 and FFA2/GPR43 receptors; and (**3**) 2-alkylthiopyranopyrimidinediones (S-alkyl derivatives of pyranopyrimidines), as potential ligands of all three aforementioned receptors. 

The HCA2/GPR109A, FFAR3/GPR41, and FFA2/GPR43 receptors are co-expressed in different types of immune cells, such as monocytes, macrophages, and neutrophils, where they reduce inflammatory function by inhibiting proinflammatory cytokine secretion and chemotaxis [30,31]. Therefore, we studied putative effects of compounds belonging to the groups **1**, **2** and **3** on the proinflammatory cytokine levels in macrophages.

The in vivo biological activity of many compounds is associated with their participation in and the effect on free radical reactions. Therefore, we evaluated the electron-donating properties of our synthesized compounds by their electro-oxidation potentials. 

The FFA receptor ligands mediate their protective effect on human brain endothelial cells through targeting the blood-brain barrier. For this reason, we calculated the topological polar surface area (TPSA) which helped us to predict the blood-brain barrier penetration ability for each compound [32]. We used calculated log *p* values to evaluate the lipophilicity of the studied compounds.

Our study has allowed us to characterise the similarity in the action by test compounds on the short-chain fatty acid receptors FFA2/GPR43, FFA3/GPR41, and to provide a comparison of their activity towards the hydroxycarboxylic acid receptor HCA2/GPR109A. Hexahydroquinoline derivatives were mainly selective and active on the FFA3/GPR41 receptor in the low micromolar range, but weaker activity towards the FFA2/GPR43 and/or HCA2/GPR109A receptors can also be manifested. The investigated oxo- and thioxopyranopyrimidines not only served as HCA2/GPR109A ligands but also stimulated the FFA3/GPR41 receptor, as well as the FFA2/GPR43 receptor with lower potency. 

The studied compounds showed anti-inflammatory properties in macrophages by reducing the proinflammatory cytokine and chemokine levels; thus, an additional argument can be made that these ligands could possess pharmacological multitarget properties to treat such complex medical conditions as cancer, Alzheimer’s disease, Parkinson’s disease, and diabetes. 

## 2. Results and Discussion

### 2.1. Synthesis of Compounds—Putative Ligands

#### 2.1.1. The Synthesis of 2-Methyl-5-oxo-4-(Aryl, Heteryl)-1,4,5,6,7,8-Hexahydroquinoline (HHQ) Derivatives **1-1**–**1-14**

**Scheme 1:**
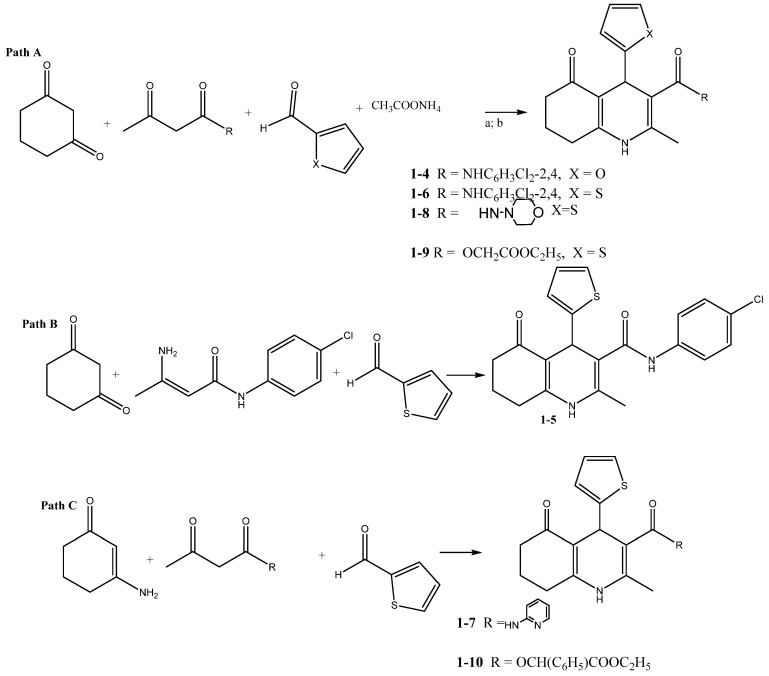
General methods for the synthesis of oxo-hexahydroquinoline derivatives. **1**. Reagents and conditions. Path A: (a) Heating without solvent at 155–160 °C, 10 min; (b) EtOH, cat. AcOH, 24 h, rt. Path B: MeOH, 24 h, rt. Path C: EtOH, 7 h, 60 °C, 24 h, rt.

The HHQ derivatives were prepared by three- or four- component Hantzsch-type cyclocondensation reactions according to the paths A, B or C (Scheme 1), using cyclohexane-1,3-dione or 3-aminocyclohex-2-en-1-one, aryl- or heterocyclic carbaldehydes, and the appropriate 3-oxo-alkanoates or 3-oxo-carboxamides, or anilides.

#### 2.1.2. The Synthesis of 5-(4-Chlorobutyl)-1H-Pyrano[2,3-d]Pyrimidine-2,4,7-Triones (Pyranopyrimidinetriones) 2–5 and 2–7, 5-Alkyl-2-Thioxo-2,3-Dihydro-4H-Pyrano[2,3-d]Pyrimidine-4,7(1H)Diones (Thioxopyranopyrimidinediones) 2-3, 2-4, 2-6 and 2(Substituted thio)-5-Alkyl-4H-Pyrano[2,3-d]Pyrimidine(1H)-Diones (2-Alkylthiopyranopyrimidinediones) 3-1–3-7

**Scheme 2:**
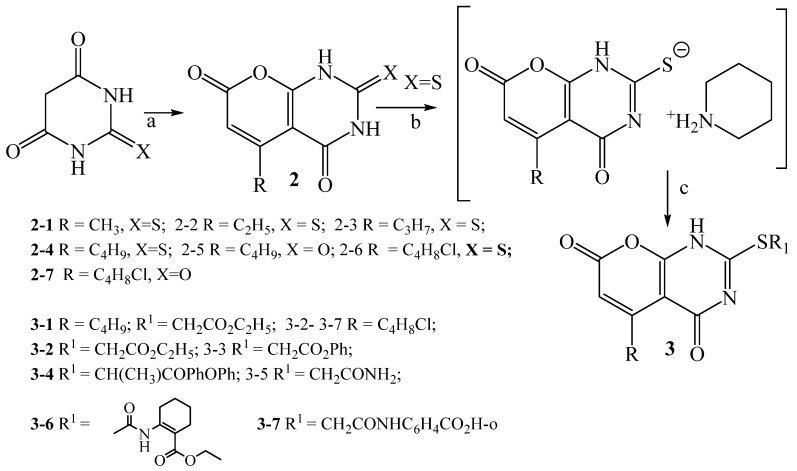
The synthetic route for obtaining pyranopyrimidines **2** and S-alkyl derivatives of pyranopyrimidines **3**. Reagents and conditions: (a) 3-oxoalkanoate (1.3 eq), glacial AcOH, 115 °C, 27 h; (b) Piperidine (1.01 eq), EtOH, reflux, 10 min; (c) Alkyl bromide (1 eq), reflux 10 min, overnight at rt.

Pyranopyrimidinetrione **2-5** was prepared according to a published method [19] by the condensation of barbituric acid with 4-alkyl-3-oxo-carboxylic acid esters. Known thioxopyranopyrimidines **2-1** and **2-2** were synthesized according to another reported procedure [20] using thiobarbituric acid instead of barbituric acid, as in the aforementioned method. The synthetic routes for compounds **2** and **3** are presented in Scheme 2.

Further alkylation of 2-thioxopyranopyrimidines **2** (X = S) was achieved through the piperidinium salts of compound **2** as non-isolated intermediates, using alkyl bromides as alkylating agents.

### 2.2. The Impact of Oxo-Hexahydroquinolines on HCA2/GPR109A, FFA3/GPR41, and FFA2/GPR43 Receptors

The first group of compounds studied as potential ligands of the HCA2/GPR109A, FFA3/GPR41, and FFA2/GPR43 receptors were the hexahydroquinoline (HHQ) derivatives **1**. For a long time, the data regarding HHQ derivatives as FFA3/GPR41 agonists and antagonists has been very limited [20,21]. Recently, a new paper has been published about structure–activity relationship studies of tetrahydroquinolones as FFA3 modulators, mainly focused on 4-alkyl- and 4-heteryl-HHQ 3-carboxamides [22]. Compound **1-3** (Table 1) has been synthesized by Arena Pharmaceuticals, evaluated as a selective agonist of the FFA3/GPR41 receptor [21,22], and proposed as a potential therapeutic agent for hepatocellular carcinoma. In order to compare our compounds to literature data, we re-synthesized three previously reported compounds (**1-1**, **1-2**, **1-3**) according to published procedures [21,22], motivated by the reported experimental data regarding their activity against one receptor (FFA3/GPR41). There are no data on these compounds regarding the FFA2/GPR43 and HCA2/GPR109A receptors. The HHQ derivatives **1-11**, **1-12**, and **1-14** are known compounds that have been synthesized as putative tools for inducing the degradation of transforming growth factor-beta receptor type II [33] (thus, a quite different type of biological activity) and were obtained according to the described procedures.

Compound **1-13** was synthesized as one of several early examples of HHQ derivatives [34], without any data on biological activity. Thus, practically no comparative data are available about the biological activity of HHQ derivatives as potential ligands of the free fatty acid receptors FFA3/GPR41 and FFA2/GPR43 and the hydroxycarboxylic acid receptor HCA2/GPR109A.

**Figure 2:**
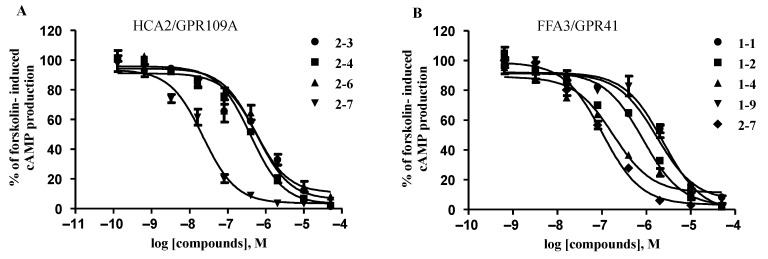
Inhibition of forskolin-stimulated cAMP production in cells expressing the HCA2/GPR109A (**A**) and FFAR3/GPR41 (**B**) receptors. Compounds were added at the indicated concentrations to measure their inhibition of forskolin-stimulated cAMP production. The curve represents the mean ± SD from two independent experiments performed in triplicate.

All of our synthesized compounds were evaluated in forskolin-stimulated cAMP accumulation assay using Flp-In-293 cells stably expressing human FFA2/GPR43, FFA3/GPR41 or HCA2/GPR109A receptors (Table 1, Table 2 and Table 3; Figure 2). The activity was described as EC_50_ values for high potency compounds and as inhibition (%) of forskolin-stimulated intracellular accumulation of cAMP by 50 µM of tested compounds in cases of lower potency.

As shown in Table 1, the group of 5-oxo-1,4,5,6,7,8-hexahydroquinoline derivatives showed selectivity towards the FFA3/GPR41 receptor; however, structural variations led to significant changes in the activity of the compounds. For example, 2,4-dichloroanilide **1–4** showed similar potency (EC_50_ = 0.23 ± 0.07 µM) to the 2,5-dichloroanilide **1–3** (EC_50_ = 0.32 ± 0.05 µM), while 4-chloroanilide **1–5** exhibited decreased activity, and for 2-pyridylamide **1–7** the activity towards FFA3/GPR41 was lost in the cAMP assay. Moreover, compound **1-7** showed activity towards FFA2/GPR43 (Table 1). In contrast, the 5-oxo-4-thienyl-1,4,5,6,7,8-hexahydroquinoline ester derivative **1-9** retained potency (EC_50_ = 1.9 ± 0.4 µM) for the FFA3/GPR41, but also was able to inhibit forskolin- stimulated cAMP through FFA2/GPR43, albeit with lower potency. At the same time, 4-*i*-butylhexahydroquinoline methyl ester described in the literature (compound **28** in [22]) showed no activity in the cAMP assay. The observed potencies of compounds **1-1** and **1-2** matched the published data [22], but there was a difference in the case of compound **1-3**.

According to [22], esters have lower activities than amides towards FFA3/GPR41; nevertheless, we demonstrated significant activity of ester **1-9**. Furthermore, in the case of the already known compounds **1-11**, **1-12**, and **1-14** the activity towards the novel receptors HCA2/GPR109A, FFA3/GPR41, and FFA2/GPR43 was tested. 

Modified compounds **1-11** and **1-14**, featuring carboxylate ester groups at position 3, had low to no activity towards the FFA3/GPR41 receptor. Compound **1-8**, possessing a morpholide group, showed a low impact on FFA3/GPR41 and lacked activity towards the FFA2/GPR43 and HCA2/GPR109A receptors. Compound **1-12**, with an *N*-alkyl lactam ring instead of an alkylamide group, had low activity towards all three studied receptors. Thus, several compounds exhibited comparable activities towards all three receptors, such as compound **1-11** (having a propylaminomethyl group at position 2 of the hexahydroquinoline system), as well as compounds **1-12** and **1-13**. Compound **1-14** (featuring a carboxyl group at position 2 of the HHQ system) completely lacked activity towards all three receptors. All three compounds (**1-11, 1-12**, **1-14**) had a biphenyl system at position 4; the same system is also part of compounds **5** and **7** [22], and the combination of a biphenyl substituent with *o*-toluidide functionality led to the highest activity for the FFA3/GPR41 receptor in the studied set of compounds. At the same time, hexahydroquinoline-3-carboxylic acid anilide (compound **1-1**) lacked activity towards FFA2/GPR43. On the other hand, hexahydroquinoline-3-carboxylic acid esters (compounds **1-9** and **1-11**) showed activity towards FFA2/GPR43 and HCA2/GPR109A. Compound **1-10** had reduced activity towards FFA3/GPR41 compared to compound **1-9** due to the additional phenyl group, but it had additional low activity towards the HCA2/GPR109A receptor.

The calculated logP values showed moderate lipophilicity. Almost all compounds had logP values below five (similarly to [22]) and TPSA less than 90: see comp. **1-1** and **4 [22]**; comp. **1-2** and **1 [22]**; comp. **1-3** and **16** [22]. 

Some tetrahydroquinolone compounds have been described as allosteric modulators of the FFA3/GPR41 receptor that can increase or decrease the potency of endogenous ligand propionate [22]. Therefore, the ability of compounds **1-1**, **1-2**, **1-3**, and **1-4** to decrease forskolin-stimulated cAMP levels was tested in the presence or absence of 5 µM propionate. The potency of compound **1-2** in the absence or presence of propionate was the same (EC_50_ = 0.61 ± 0.09 µM and EC_50_ = 0.58 ± 0.03 µM, respectively), which was in a good agreement with the potency reported by Ulven et al. [22] for compound **1** (EC_50_ = 0.52 µM and EC_50_ = 0.54 µM, respectively). The potency of compound **1-1** (EC_50_ = 1.79 ± 0.3 µM) was slightly increased in the presence of propionate (EC_50_ = 0.64 ± 0.2 µM), with values close to those reported for compound **4** [23]. The potencies of compounds **1-3** (EC_50_ = 0.32 ± 0.05 µM) and **1-4** (EC_50_ = 0.23 ± 0.07 µM) were increased in the presence of propionate (EC_50_ = 0.10 ± 0.02 µM and EC_50_ = 0.081± 0.04 µM, respectively). These results suggest that compounds **1-1**, **1-3**, and **1-4** could stimulate the FFA3/GPR41 receptor as agonists and potentiate the activity of propionate.

### 2.3. The Effects of Pyranopyrimidine Derivatives on HCA2/GPR109A, FFA2/GPR43, and FFA3/GPR41 Receptors

Pyranopyrimidines have been tested so far only as HCA2/GPR109A receptor ligands [19,20]. We studied the possible activities of pyranopyrimidines towards three GPCRs: HCA2/GPR109A, FFA2/GPR43, and FFA3/GPR41. Besides the newly synthesized compounds, we studied two known compounds (**2-2** and **2-5**) for which literature data were available concerning their activity towards one receptor (HCA2/GPR109A). Some structure–activity relationships of the studied compounds (Table 2) have been identified. We observed two main types of ligand–receptor potency patterns.

1. High potency for HCA2/GPR109A, low potencies for FFA3/GPR41 and FFA2/GPR43: compounds **2-3** and **2-5** (5-*n*-propyl-2-thioxo and 5-*n*-butyl-2-oxo derivatives of pyranopyrimidines).
2. High and comparable potencies towards all three studied receptors: compounds **2-1**, **2-2**, **2-4**, **2-6**, and **2-7**, representing 2-thioxo- and 2-oxopyranopyrimidines.

This activity pattern can be divided into subtypes: in four cases, the compounds were more active towards HCA2/GPR109A, but in one case compound **2-4** (a derivative of 2-thioxo-5-butylpyranopyrimidine) was more active towards FFA3/GPR41 than HCA2/GPR109A.

Thus, in principle, either multitarget or selectively active compounds possessing different selectivity could be designed. The prolongation of the alkyl chain (propyl → butyl) enhanced activity towards all studied receptors (according to the comparison of compounds **2-3** and **2-4**). We added a novel 2-oxopyranopyrimidine derivative to the group of compounds described by Palani et al. [18,19,35]: 5-chlorobutyl-2-oxopyranopyrimidine (compound **2-7**). It had the same activity on the HCA2/GPR109A receptor as its butyl analogue, but the impact on FFA3/GPR41 and FFA2/GPR43 was significantly increased compared to the 5-butyl compound **2-5**.

The potency trends were different in the group of 2-thioxopyranopyrimidines. The 5-chlorobutyl derivative **2-6** was more potent towards HCA2/GPR109A receptor, but less potent towards FFA3/GPR41 and FFA2/GPR43 receptors, compared to the 5-butyl derivative **2-4**.

Compound **2-7** (possessing chlorobutyl substituent) had the highest activity (in the group of compounds **2-3**, **2-4**, **2-6**, and **2-7**) in inhibition of forskolin-stimulated cAMP production (Figure 2A). Compound **2-7** was also the most active compound compared with the hexahydroquinoline type compounds **1-1**, **1-2**, **1-4**, and **1-9**.

Evidently, compounds of this type provide an appropriate platform for further optimization in the most preferable direction.

The observed potency of compound **2-3** matched literature data [19], but there was a difference in the case of compound **2-5** [18].

### 2.4. The Impact of S–Alkylpyranopyrimidine Derivatives on HCA2/GPR109A, FFA3/GPR41, and FFA2/GPR43 Receptors

*S*-Alkylpyranopyrimidine derivatives had not yet been studied as ligands of short-chain fatty acid or hydroxycarboxylic acid receptors. Remarkably, the aforementioned compounds were shown to have activity towards all three receptors, especially the FFA3/GPR41 and HCA2/GPR109A receptors (Table 3).

*2-Ethoxycarbonylmethyl derivatives***3-1***and***3-2**: increased potency towards FFA3/GPR41, less potency towards HCA2/GPR109A, and the least activity towards FFA2/GPR43.

*2-Benzyloxycarbonylmethyl derivative***3-3**: phenyl group instead of methyl group (comp. **3**-**2**) led to a decrease of activity towards FFA3/GPR41, no change in activity towards FFA2/GPR43, but an increase in potency towards HCA2/GPR109A.

*2-Benzyloxybenzoylethyl derivative***3-4:** a compound possessing two phenyl rings and a ketone carbonyl group instead of an ester group had the highest potency towards FFA3/GPR41, but lower activities towards FFA2/GPR43 and HCA2/GPR109A.

*Carbamoylmethyl derivatives***3-5**–**3-7**: these compounds in general had a similar or weaker impact on all three receptors compared to the ester or ketone carbonyl compounds **3-1**–**3-4**. This trend was especially pronounced for potencies towards FFA3/GPR41; EC_50_ was substantially increased (compounds **3-6** and **3-7** versus compounds **3-2** and **3-4**).

The studied compounds had a remarkable potency (EC_50_ from 0.38 to 8.2 µM for selected compounds) towards FFA3/GPR41 albeit not reaching the high potencies of the aforementioned oxo- or thioxopyranopyrimidines (Table 2), but exceeding their potency towards HCA2/GPR109A (EC_50_ from 17 to 75 µM or even lower concentration). In the case of activity towards the FFA2/GPR43 r, it was not possible to determine EC_50_, which was in some cases also true for FFA3/GPR41.

It can be concluded that *S*-alkylpyranopyrimidines are more selective for the FFA3/GPR41 and HCA2/GPR109A receptors than for the FFA2/GPR43 receptor.

The activity of oxo- and thioxopyranopyrimidines towards the studied receptors was different (Table 2), as the mentioned ligands preferentially had more impact on HCA2/GPR109A receptor, but *S*-alkylpyranopyrimidines preferentially had more impact on FFA3/GPR41 receptor (Table 3). Such knowledge could be useful for further development of specific and multitarget ligands. 

Compounds that were not able to change the forskolin-stimulated cAMP level were tested for their antagonist activity in the presence of the respective receptor agonist. None of these compounds were able to change the agonist activity (data not shown).

In general, it is possible to obtain compounds possessing high potencies on one, two or three receptors, or high selective activity on one out of three receptors.

### 2.5. Electron-Donating Properties of Hexahydroquinolines and Pyranopyrimidines

The electron-donating properties of the studied series of compounds were characterised by their electrochemical oxidation potentials. Derivatives of 1,4,5,6,7,8-hexahydroquinoline are bicyclic analogs of 1,4-dihydropyridines, well-known partially hydrogenated heterocyclic compounds, which in turn are analogs of 1,4-dihydronicotinamides and can be used as model compounds of the redox coenzyme NAD(P)H. 1,4-Dihydropyridines have shown antiradical and antioxidant activity [37,38,39,40].

4-Aryl(hetaryl) derivatives of 3,5-dialkoxycarbonyl-1,4-DHP are well-known Ca^2+^ channel antagonists. Several Ca^2+^ antagonists (such as nitrendipine, amlodipine, nifedipine, felodipine) possess antioxidant activity [41]. DHP derivatives nisoldipine and amlodipine diminish superoxide formation [42].

The antioxidant activity of dihydropyridines was subsequently correlated with different biological and pharmacological properties of these compounds [43,44]. The antioxidant activity of DHP derivatives was characterised using electrochemical methods [45].

The antioxidant activity of fatty hexahydroquinolines, as well as hexahydroquinoline derivatives has been described [46,47,48].

Compounds containing fatty chains as substituents at the positions 2 or 3 and an aryl moiety at position 4 have good antioxidant activity, approaching even that of the classic antioxidants BHT (butylated hydroxytoluene) and vitamin E.

The electron-donating properties of the studied types of compounds were revealed by electrochemical methods. The hexahydroquinoline derivatives **1-1, 1-2, 1-4, 1-9** and **1-10** had electrooxidation potentials at 1.18 V–1.24 V and 1.68 V–1.99 V (Table 1). The lower electrooxidation potentials correspond to electron donor property.

Pyranopyrimidines **2-5** and **2-7** had electrooxidation potentials of 1.94 V (for both compounds (Table 2). The electrooxidation potentials of *S*-alkylpyranopyrimidines were at 1.93 V–1.98 V (Table 3). These compounds lack electron donor properties.

### 2.6. Lipophilicity and Polar Surface Area of Studied Compounds

The lipophilicity and polar surface area of the three studied groups of compounds were calculated as basic molecular characteristics pertaining to the potential medicinal use of these compounds.

Almost all of the synthesized and studied compounds had acceptable lipophilicity for peroral bioavailability (logP < 5) [49]. According to the calculated values, even lower lipophilicity was observed, thus a broader range of functionalities could be included in similar compounds during further structural optimization in order to design potential drug molecules for injectable final dosage forms [50].

Short-chain fatty acids have strong effects on neuronal physiology. The free fatty acid receptors FFA2/GPR43 and FFA/GPR41 are expressed in the brain [51,52] and play a significant role in human health conditions. The TPSA (topological polar surface area, Å^2^) values characterise the ability of molecules to cross the blood-brain barrier. The calculated TPSA values for derivatives of hexahydroquinoline, oxopyranopyrimidines, and thioxopyranopyrimidines were estimated to mostly be lower than 90 Å^2^ (Table 1 and Table 2), while the calculated TPSA values for alkylmercaptopyranopyrimidines ranged from 97 to 127 Å^2^ or even 135 Å^2^ (Table 3), making these compounds in general appropriate for transport through cell membranes, and in most cases also through the blood-brain barrier [53].

### 2.7. Ligands of HCA2/GPR109A, FFA3/GPR41, and FFA2/GPR43 Receptors Suppress Gene Expression and Secretion of Inflammatory Cytokines in THP-1 Macrophages

**Figure 3:**
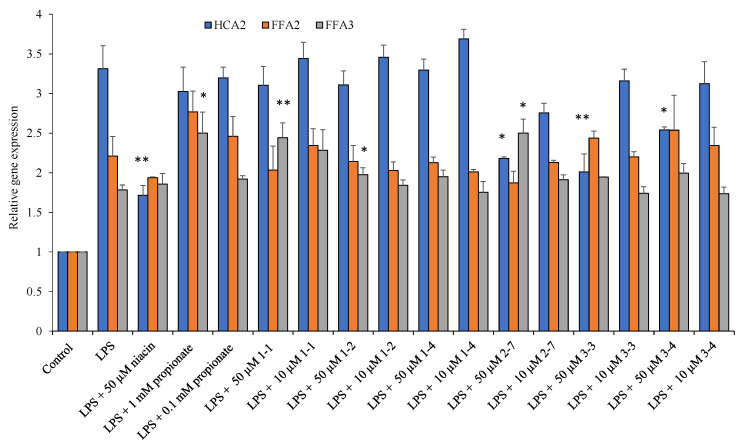
The effect of LPS and test compounds on gene expression for the HCA2/GPR109A, FFAR3/GPR41, and FFA2/GPR43 receptors in THP-1 macrophages. The cells were treated for 4 h with LPS (1 µg/mL) in the presence or absence of test compounds at various concentrations. The gene expression for the receptors was analysed by real-time PCR and is reported relative to the value from untreated control cells, which was defined as 1. The data were normalized to *RPS29* and the values were calculated using the comparative (2^−ΔCt^) method. The results are given as the mean ± SD from three independent experiments. * *p* < 0.05, ** *p* < 0.01 are significantly different from LPS-stimulated cells.

Besides the studies focused on the activity of compounds on the free fatty acid receptors FFA3/GPR41 and FFA2/GPR43 and the hydroxycarboxylic acid receptor HCA2/GPR109A we took into consideration the co-expression of HCA2/GPR109A, FFA3/GPR41, and FFA2/GPR43 in immune cells (monocytes, macrophages, neutrophils), where they reduce proinflammatory function [54]. In order to investigate the biological activities of compounds **1**, **2**, **3**, we stimulated human THP-1 macrophages with the test compounds at 10 µM and 50 µM concentrations, 50 µM of the HCA2/GPR109A selective ligand niacin, as well as 0.1 mM and 1 mM of the FFA2/GPR43 and FFAR3/GPR41 ligand propionate. Compounds **1-1**, **1-2** and **1-4** were tested as FFA3/GPR41 selective compounds, while compounds **2-7**, **3-3**, and **3-4** were tested as compounds lacking selectivity for that receptor. Compounds **1-1** and **1-2** are well-known representatives of the hexahydroquinoline group of FFAR3/GPR41 ligands, having an *o*-toluidide side chain at position 3 and 4-thienyl or 4-furyl group at position 4, and compound **1-4** is a novel active ligand of FFAR3/GPR41, possessing a 2,4-dichloroanilide substituent at position 3, representing an isomer of the known 2,5-dichloroanilide compound **1-3**. Concerning the derivatives of pyranopyrimidines, we studied interesting 5-chlorobutyl derivatives: compound **2-7** as a representative of 2-oxopyranopyrimidines and two *S*-alkylthiopyranopyrimidines, **3-3** and **3-4**, bearing ester and benzoylethyl moieties. First, we examined the gene expression of all three receptors in unstimulated and 4 h LPS-stimulated human THP-1 macrophages. Figure 3 shows that all three receptors were expressed in THP-1 cells, with higher HCA2/GPR109A levels than FFA2/GPR43 and FFA3/GPR41. LPS stimulation of cells markedly increased the gene expression for all three receptors compared to unstimulated cells. Niacin significantly reduced LPS upregulation of the HCA2/GPR109A (*p* < 0.01), but did not affect the expression of FFA2/GPR43 and FFA3/GPR41 genes. Compounds **2-7**, **3-3** and **3-4** at 50 µM concentration also significantly reduced the LPS-stimulated HCA2/GPR109A mRNA levels (*p* < 0.05), resembling the effect of niacin. In contrast, the LPS-upregulated FFA2/GPR43 and FFA3/GPR41 gene expression was slightly increased by the addition of 1mM propionate and some of the compounds; however, this effect was not statistically significant compared to the LPS-stimulated cells (*p* > 0.05) (Figure 3). Propionate at 1 mM and compounds **1-1**, **1-2**, **2-7** and **3-3** at 50 µM concentrations significantly increased the LPS-stimulated FFA3/GPR41 mRNA levels (*p* < 0.05).

**Figure 4:**
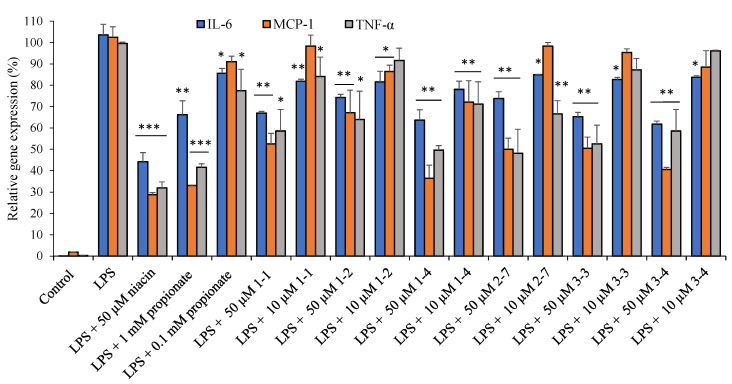
The modulation of LPS-induced proinflammatory cytokine and chemokine gene expression by ligands of the HCA2/GPR109A, FFA3/GPR41, and FFA2/GPR43 receptors in THP-1 macrophages. The cells were treated for 4 h with LPS (1 µg/mL) in the presence or absence of various concentrations of compounds. The levels of the cytokines IL-6 and TNF-α and the chemokine MCP-1 mRNA were determined by real-time PCR and expressed relative to the value from LPS-treated cells, defined as 100%. The data were normalized to *RPS29* and the values were calculated by using the comparative (2^−ΔCt^) method. The results are reported as the mean ± SD from at least three independent experiments. * *p* < 0.05, ** *p* < 0.01, *** *p* < 0.0001 are significantly different from LPS-stimulated cells.

Further, we quantified the gene expression levels of the pro-inflammatory cytokines IL-6 and TNF-α and the chemokine-MCP-1 in untreated, LPS-treated, and LPS/test compound-treated THP-1 macrophages, respectively. The expression of each molecule at the mRNA level was significantly upregulated after 4 h stimulation with LPS in THP-1 cells (Figure 4). The HCA2/GPR109A ligand niacin (50 µM), FFA2/GPR43 and FFA3/GPR41 ligand propionate (1 mM) induced a significant downregulation of the LPS-induced IL-6 (44 ± 4, *p* < 0.0001 and 66 ± 5%, *p* < 0.001), MCP-1 (29 ± 1 and 33 ± 2%, *p* < 0.001), and TNF-α (32 ± 3 and 42 ± 4%, *p* < 0.001), respectively. Furthermore, all tested compounds reduced cytokine gene expression in a dose-dependent manner. A significant decrease in IL-6 mRNA (67 ± 1, 74 ± 1, and 64 ± 5%, *p* < 0.01), TNF-α mRNA (59 ± 10, 64 ± 13, *p* < 0.05, and 50 ± 2%, *p* < 0.001), and MCP-1 mRNA levels (53 ± 5, 67 ± 10, and 37 ± 6%, *p* < 0.01) was detectable at 50 µg/mL for compounds **1-1**, **1-2** and **1-4**, respectively. The non-selective compounds **2-7**, **3-3** and **3-4** at 50 µg/mL concentration also showed a significant decrease in IL-6 mRNA (74 ± 3, 65 ± 2, and 61 ± 1%, *p* < 0.001), TNF-α mRNA (48 ± 11, 53 ± 9, and 58 ± 10%, *p* < 0.001), and MCP-1 mRNA levels (50 ± 5, 49 ± 5, and 41 ± 1%, *p* < 0.001), respectively (Figure 4). 

**Figure 5:**
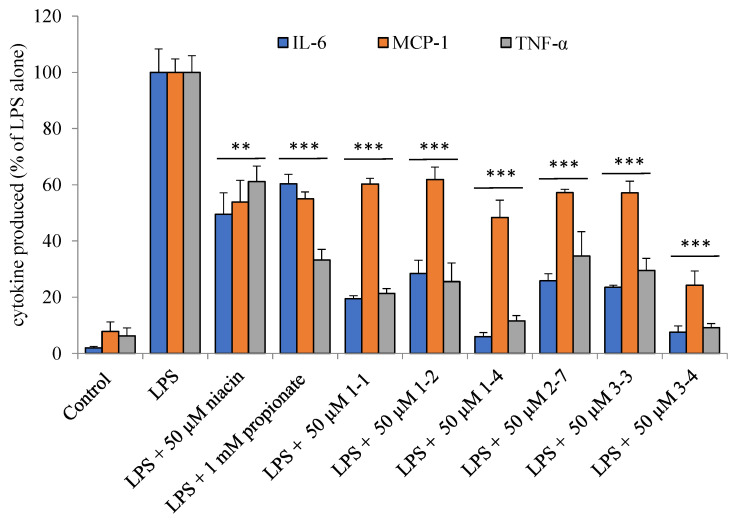
The suppression of pro-inflammatory cytokine and chemokine secretion from THP-1 macrophages after treatment with the test compounds. Supernatants from THP-1 macrophages treated for 4 h with LPS and test compounds were subjected to Luminex assay to detect the levels of the pro-inflammatory cytokines IL-6 and TNF-α and the chemokine MCP-1. Results are presented as the mean values ± SD from three independent experiments, where ** *p*< 0.01, *** *p* < 0.0001 are significantly different from LPS-stimulated cells.

As the next step, we measured cytokine levels secreted into the medium when THP-1 macrophages were exposed to LPS (1 µg/mL) in the presence or absence of compounds (50 µg/mL). After 4 h, the culture medium was collected and cytokine concentrations were measured with a Luminex kit. Cytokine secretion significantly increased following macrophage treatment with LPS (Figure 5). All of the compounds were able to significantly reduce LPS-stimulated secretion of IL-6, TNF-α and MCP-1 from macrophages (Figure 5).

**Figure 6:**
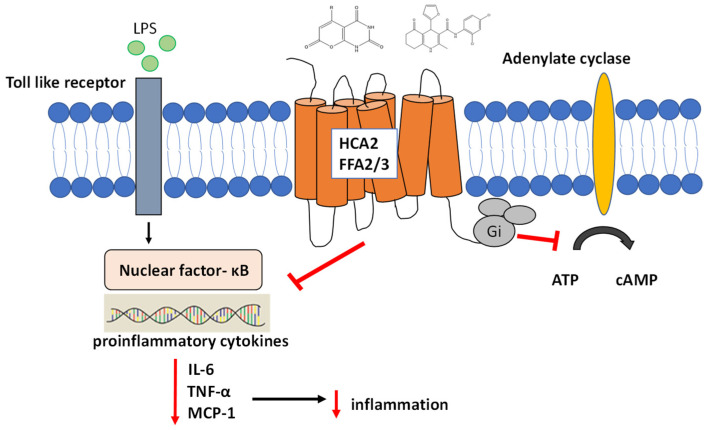
Schematic representation of the effects of studied compounds mediated in cells expressing FFA2/3 and HCA2 receptors.

The results showed that the activation of HCA2/GPR109A, FFA3/GPR41, and FFA2/GPR43 by the studied compounds can reduce the proinflammatory cytokine levels in macrophages, which would be beneficial in treating inflammatory conditions (Figure 6). Macrophages produce proinflammatory cytokines through the activation of toll-like receptors and the nuclear factor κB (NF- κB) pathway, with subsequent regulation of inflammatory genes. It has been shown that the FFA and HCA2 receptors mediated signaling pathways suppress NF- κB signalling in lung tissues and macrophages [6,12]. The observed inhibition of proinflammatory cytokines in macrophages by the studied compounds can be mediated via inactivation of NF- κB; however, the precise mechanism should be further elucidated.

Our findings generally agree with the recent publications about multitarget pleiotropic drugs including 1,4-DHPs [55] and their usefulness as modulators for GPCR (especially anti-inflammatory SCFA receptors) for the treatment of Diabetes mellitus (T1D) [56]. 

## 3. Materials and Methods

### 3.1. Materials

All materials for cell cultures were supplied by Thermo Fisher Scientific. A Lance cAMP kit was purchased from PerkinElmer. The pcDNA3.1-FFA2R and pcDNA3.1-FFA3R plasmids were obtained from cDNA Resource Center (www.cdna.org; accessed on 20 June 2020). All other compounds and reagents were purchased from Acros Organics, Sigma-Aldrich or Alfa Aesar, unless stated otherwise, and were used without further purification.

### 3.2. Synthesis

#### 3.2.1. Hexahydroquinolines

Hexahydroquinolines **1-1**, **1-2**, **1-3**, **1-11**, **1-12**, **1-13** and **1-14** have been reported in the literature (references given in Table 1), with ^1^H and ^13^C NMR spectra of the synthesized samples matching the published data.

##### General Procedure for the Synthesis of **5**-Oxo-hexahydroquinolines **1-4**, **1-6**, **1-8**, and **1-9**

Path A

(a) A mixture of cyclohexane-1,3-dione (0.56 g, 5 mmol), the appropriate aldehyde (5 mmol), the appropriate anilide (5 mmol), and ammonium acetate (1.2 g, 15 mmol) was stirred and heated under solvent-free conditions at 155-160 °C temperature until the mixture solidified (10-15 min). The mixture was triturated with water and the precipitate was collected by filtration. The crude product was purified by silica gel column chromatography using 1:2 ethyl acetate/petroleum ether as eluent, to afford compounds **1-4**, **1-6**.

(b) A mixture of cyclohexane-1,3-dione (0.56 g, 5 mol), the appropriate aldehyde (5 mmol), the appropriate alkyl 3-oxobutanoate or anilide (5 mmol), and ammonium acetate (1.2 g, 15 mmol) in ethanol (15 mL) was treated by adding five drops of acetic acid, followed by stirring at rt for 24 h. The precipitate that formed upon cooling in a refrigerator was collected by filtration, then washed with water and cold ethanol to afford compounds **1-8**, **1-9**.

##### *N*-(2,4-Dichlorophenyl)-4-(furan-2-yl)-2-methyl-5-oxo-1,4,5,6,7,8-hexahydroquinoline-3-carboxamide **1-4**

Compound **1-4** was prepared according to the general procedure (Path **A, a**) from cyclohexane-1,3-dione (0.56 g, 5 mmol), furan-2-carbaldehyde (0.41 mL, 5 mmol), *N*-(2,4-dichlorophenyl)-3-oxobutanamide (1.35 g, 5.5 mmol), and ammonium acetate (0.45 g, 5.8 mmol). Yield of compound **1-4**: (810 mg, 39%) as light yellow foam. ^1^H NMR (400 MHz, CDCl_3_): 8.26-8.12 (m, 2H), 7.36–7.29 (m, 2H), 7.20 (dd, *J* = 8.7, 2.4 Hz, 1H), 6.28 (dd, *J* = 3.2, 1.8 Hz, 1H), 6.20-6.12 (m, 1H), 6.05 (s, 1H), 5.15 (s, 1H), 2.55–2.40 (m, 4H), 2.35 (s, 3H), 2.05 (d, *J* = 18.3 Hz, 2H). ^13^C (CDCl_3_): 195.07, 165.95, 156.06, 150.64, 143.47, 142.17, 134.08, 128.74, 128.70, 127.49, 123.97, 123.02, 110.57, 109.36, 106.46, 105.02, 36.83, 30.67, 27.55, 21.02, 19.34. MS (ES+), *m/z*: 418 ([M+H]^+^, 100). Calculated for C_21_H_18_ Cl_2_N_2_O_3_: C, 60.45; H, 4.35; N, 6.71; Found: C, 60.10; H, 4.37; N, 6.66.

##### *N*-(2,4-Dichlorophenyl)-2-methyl-5-oxo-4-(thiophen-2-yl)-1,4,5,6,7,8-hexahydroquinoline-3-carboxamide **1-6**

Compound **1-6** was prepared according to the general procedure (Path **A, a**) from cyclohexane-1,3-dione (0.56 g, 5 mmol), thiophene-2-carbaldehyde (0.56 g, 5 mmol), *N*-(2,4-dichlorophenyl)-3-oxobutanamide (1.35 g, 5.5 mmol), and ammonium acetate (0.45 g, 5.8 mmol). Yield of compound **1-6**: (760 mg, 35%) as light yellow powder. Mp: >200 °C. ^1^H NMR (400 MHz, DMSO-*d*_6_): 9.11 (s, 1H), 8.96 (s, 1H), 7.72 (d, *J* = 8.8 Hz, 1H), 7.60 (d, *J* = 2.4 Hz, 1H), 7.38 dd, *J* = 8.7, 2.4 Hz, 1H), 7.23 (dd, *J* = 5.1, 1.3 Hz, 1H), 6.88-6.75 (m, 2H), 5.24 (s, 1H), 2.48-2.46 (m, 2H), 2.32-2.13 (m, 5H), 1.93 (dt, *J* = 13.0, 4.8 Hz, 2H). ^13^C NMR (DMSO-*d*_6_): 198.30, 167.06, 152.35, 151.27, 140.06, 135.08, 129.40, 129.16, 127.94, 127.91, 126.99, 124.66, 123.83, 110.03, 106.00, 37.16, 32.61, 26.69, 21.24, 16.12, 14.56. MS (ES+), *m/z*: 434 ([M+H]^+^, 100). Calculated for C_21_H_18_ Cl_2_N_2_O_2_S: C, 58.21; H,4.19; N, 6.46; Found: C, 57.89; H, 4.17; N, 6.40.

##### 2-Methyl-3-(morpholine-4-carbonyl)-4-(thiophen-2-yl)-4,6,7,8-tetrahydro-1*H*-quinolin-5-one **1-8**

Compound **1-8** was prepared according to the general procedure (Path **A, b**) from cyclohexane-1,3-dione (0.56 g, 5 mmol), thiophene-2-carbaldehyde (0.56 g, 5 mmol), 1-morpholinobutane-1,3-dione (0.86 g, 5mmol), and ammonium acetate (1.2 g, 15 mmol). Yield of compound **1-8**: 787 mg (44%) as light yellow powder. Mp: 178 °C. ^1^H NMR (400 MHz, DMSO-*d*_6_): 8.75 (s, 1H), 7.19 (dd, *J* = 9.0, 2.4 Hz, 1H), 6.82 (dd, *J* = 8.9, 3.4 Hz, 1H), 6.62 (m, 1H), 4.90 (s, 1H), 3.28 (s, 3H), 2.44 (m, 8H), 2.16 (m, 2H), 1.86 (m, 2H), 1.69 (s, 2H). MS (ES+), *m/z*: 359 ([M+H]^+^, 100). Calculated for C_19_H_22_N_2_O_3_S: C, 63.66; H,6.19; N, 7.81; Found: C, 63.54; H, 6.26; N, 7.70.

##### Ethoxycarbonylmethyl 2-Methyl-3-(morpholine-4-carbonyl)-4-(thiophen-2-yl)-4,6,7,8-tetrahydr **1-9**

Compound **1-9** was prepared according to the general procedure (Path **A, b**) from cyclohexane-1,3-dione (0.56 g, 5 mmol), thiophene-2-carbaldehyde (0.56 g, 5 mmol), (2-ethoxy-2-oxoethyl) 3-oxo-butanoate (0.94 g, 5 mmol), and ammonium acetate (1.2 g, 15 mmol). Yield of compound **1-9**: 600 mg (37%) as light yellow powder, Mp: 150°C. ^1^H NMR (400 MHz, DMSO-*d*_6_): 9.43 (s, 1H), 7.16 (d, *J* = 5.0 Hz, 1H), 6.82 (m, 1H), 6.71 (m, 1H), 5.20 (s, 1H), 4.68 (dd, *J* = 15.6, 7.4 Hz, 2H), 4.1 (q, *J* = 7.0, 2H), 2.52–2.46 (m, 2H), 2.28 (s, 3H), 2.32–2.25 (m, 2H), 1.98–1.77 (m, 2H), 1.76 (t, *J* = 7.0 Hz, 3H). ^13^C NMR (DMSO-*d*_6_): 195.07, 168.56, 166.53, 151.94, 147.56, 126.97, 123.77, 123.03, 111.36, 102.24, 61.04, 60.71, 37.10, 32.59, 30.56, 26.48, 26.45, 21.26, 18.83,14.42. MS (ES-), *m/z*: 374 ([M-H]^−^, 100). Calculated for C_19_H_21_NO_5_S: C, 60.78; H, 5.64; N, 3.73; Found: C, 60.50; H, 5.80; N, 3.66.

##### N-(4-Chlorophenyl)-2-methyl-5-oxo-4-(thiophen-2-yl)-1,4,5,6,7,8-hexahydroquinoline-3-carboxamide **1-5**

Path B

A mixture of cyclohexane-1,3-dione (0.56 g, 5 mmol), thiophene-2-carbaldehyde (0.56, 5 mmol), (*E*,*Z*)-3-amino-*N*-(4-chlorophenyl)but-2-enamide (1.15 g, 5.5 mmol) in methanol (10 mL) was stirred at rt for 24 h. The solvent was removed under reduced pressure and the crude product was purified by silica gel column chromatography using 1:2 ethyl acetate/petroleum ether as eluent to afford compound **1-5** (837 mg, 42%) as a light yellow foam. ^1^H NMR (400 MHz, CDCl_3_): 7.45 (s, 1H), 7.26–7.23 (m, 2H), 7.22–7.18 (m, 2H), 7.07 (dd, *J* = 3.6, 1.2 Hz, 1H), 6.95 (dd, *J* = 5.1, 3.6 Hz, 1H), 6.23–6.12 (m, 1H), 5.87 (s, 1H), 2.53–2.38 (m, 7H), 2.09-1.89 (m, 2H). MS (ES+), *m/z*: 399 ([M+H]^+^, 100). Calculated for C_21_H_19_ClN_2_O_2_S: C, 63.23; H, 4.80; N, 7.02; Found: C, 62.90; H, 4.85; N, 6.95.

##### General Procedure for the Synthesis of 5-Oxo-hexahydroquinolines **1-7**, **1-10**

Path C

A mixture of 3-aminocyclohex-2-en-1-one (0.33 g, 3 mmol), the appropriate aldehyde (3 mmol) and the appropriate 3-oxo-alkanoate or 3-oxo-carboxylic acid anilide (3 mmol) in ethanol or 2-propanol (6 mL) was stirred at 60 °C for 7 h and then at rt for 24 h. After cooling, the precipitate was collected by filtration and recrystallized from ethanol.

##### 2-Methyl-5-oxo-*N-*(pyridin-2-yl)-4-(thiophen-2-yl)-1,4,5,6,7,8-hexahydroquinoline-3-carboxamide **1-7**

Compound **1-7** was prepared according to the general procedure (**Path C**) from 3-aminocyclohex-2-en-1-one (0.33 g, 3 mmol), thiophene-2-carbaldehyde (0.34 g, 3 mmol), and 3-oxo-*N*-(pyridin-2-yl)butanamide (0.54 g, 3 mmol). The mixture in 2-propanol was refluxed for 6 h. Yield of compound **1-7**: 241 mg (22%) as a light yellow powder. Mp 239 °C. ^1^H NMR (400 MHz, CDCl_3_): 8.20 (ddd, *J* = 4.8, 1.6, 0.8 Hz, 1H), 8.14 (ddd, *J* = 7.6, 0.8, 0.8 Hz, 1H), 8.08 (bs, 1H), 7.62 (td, *J* = 7.6, 2.4 Hz, 1H), 7.14 (dd, *J* = 5.2, 1.2 Hz, 1H), 7.06 (ddd, *J* = 3.6, 1.2, 0.8 Hz, 1H), 6.95 (ddd, *J* = 7.2, 4.8, 1.2 Hz, 1H), 6.92 (dd, *J* = 5.2, 3.6 Hz, 1H), 6.19 (s, 1H), 5.36 (s, 1H), 2.40 (s, 3H), 2.50–2.30 (m, 4H), 2.06–1.89 (m, 2H). MS (ES+), *m/z*: 366 ([M+H]^+^, 100). Calculated for C_20_H_19_N_3_O_2_S: C, 65.73; H, 5.24; N, 11.50; Found: C, 65.70; H, 5.26; N, 11.43.

##### (2-Ethoxy-2-oxo-1-phenylethyl)2-methyl-5-oxo-4-(2-thienyl)-4,6,7,8-tetrahydro-1*H*-quinoline-3-carboxylate **1-10**

Compound **1-10** was prepared according to the general procedure (**Path C**) from 3-aminocyclohex-2-en-1-one (0.33 g, 3 mmol), thiophene-2-carbaldehyde (0.34 g, 3 mmol), and 2-ethoxy-2-oxo-1-phenylethyl 3-oxobutanoate (0.79 g, 3 mmol). Yield of compound **1-10**: 433 mg (32%) as a light yellow powder. Mp: 160–163 °C. ^1^H NMR (400 MHz, DMSO-*d*_6_): 7.39 (m, 2H), 7.33 (m, 3H), 7.04 (dd, *J* = 24.3, 3.1 Hz, 1H), 6.96 (m, 1H), 6.86 (dd, *J* = 8.9, 2.4 Hz, 1H)*,* 6.39 (s, 1H), 5.88 (s, 1H), 5.47 (s, 1H), 4.15–4.09 (m, 2H), 2.41–2.31 (m, 7H), 1.99–1.93 (m, 2H), 1.13 (t, *J* = 7.3 Hz, 3H). MS (ES+), *m*/*z*: 452 ([M+H]^+^, 100). Calculated for C_25_H_25_NO_5_S: C, 66.50; H, 5.58; N, 3.10; Found: C, 66.39; H, 5.67; N, 2.99.

#### 3.2.2. Pyranopyrimidines and Thioxopyranopyrimidines

Pyranopyrimidine **2-2**, as well as the thioxopyranopyrimidines **2-1** and **2-5** are known in the literature [18,19,27]. ^1^H and ^13^C NMR spectra of the obtained samples matched the published data.

##### 5-(4-Chlorobutyl)-1H-pyrano[2,3-d]pyrimidine-2,4,7-trione **2-7**

A mixture of barbituric acid (0.90 g, 7 mmol, 1 eq) and ethyl 7-chloro-3-oxo-heptanoate (1.88 g, 9.1 mmol, 1.3 eq) was combined with glacial acetic acid (15 mL) in a vial, followed by heating under argon atmosphere at 115 °C for 27 h. The hot mixture was filtered and the solids were washed with hot acetic acid. The acetic acid was removed from the filtrate under reduced pressure and the residue was triturated with hot ethyl acetate (70 mL). The precipitate was filtered and washed with hot ethyl acetate (10 mL). The solution was concentrated in vacuo. The residue was dissolved in hot DMF (3 mL); water (~4 mL) was then added dropwise until the precipitation was complete. The light grey powder was collected by filtration, recrystallized from ethanol, and dried at reduced pressure, yielding compound **2-7** (210 mg, 11%), Mp > 200 °C. ^1^H NMR (400 MHz, DMSO-*d*_6_): 12.72 (s, 1H), 11.35 (s, 1H), 5.84 (s, 1H), 3.65 (t, *J* = 6.6 Hz, 2H), 2.87 (t, *J* = 7.4 Hz, 2H), 1.77 (dq, *J* = 8.7, 6.5 Hz, 2H), 1.68-1.54 (m, 2H). ^13^C NMR (DMSO-*d*_6_): 161.64, 161.53, 161.21, 157.37, 148.85, 109.09, 91.86, 46.58, 32.85, 32.09, 26.41. MS (ES−), *m/z*: 269 ([M-H]^−^, 100). Calculated for C_11_H_11_ClN_2_O_4_: C, 48.81; H, 4.10; N, 10.35; Found: C, 48.52; H, 4.15; N, 10.12.

#####  General Procedure for the Synthesis of 5-Alkyl-2-thioxo-2,3-dihydro-4H-pyrano[2,3-d]pyrimidine-4,7(1H)-diones **2-3**, **2-4**, **2-6**

A mixture of thiobarbituric acid (1.44 g, 10 mmol, 1 eq) and the appropriate ethyl (methyl) 4-alkyl-3-oxo-alkanoate (12.6 mmol, 1.26 eq) was added to a vial with glacial acetic acid (15 mL) under argon atmosphere and the mixture was heated at 120 °C for 30 h. The hot mixture was filtered and the solids were washed with hot acetic acid. The acetic acid was removed from the filtrate under reduced pressure and the residue was triturated with hot ethyl acetate (70 mL). The precipitate was collected by filtration and washed with hot ethyl acetate (10 mL). The solution was concentrated under reduced pressure. The residue was dissolved in hot DMF (4 mL) and then water (~5 mL) was added dropwise until the precipitation was complete. The obtained light grey powder was collected by filtration, recrystallized from ethanol, and dried under reduced pressure. The obtained 2-thioxopyranopyrimidines were recrystallized from ethanol.

##### 5-Propyl-2-thioxo-2,3-dihydro-1*H*-pyrano[2,3-*d*]pyrimidine-4,7-dionee **2-3**

Compound **2-3** was prepared according to the general procedure from thiobarbituric acid (1.44 g, 10 mmol) and ethyl 3-oxohexanoate (2.03 mL, 12.6 mmol). Yield of compound **2-5**: 360 mg (15%) as light grey powder, Mp > 200 °C. ^1^H NMR (400 MHz, DMSO-*d*_6_): 12.58 (s, 1H), 5.87 (s, 1H), 2.77 (t, *J* = 7.6 Hz, 2H), 1.52-1.43 (m, 2H), 0.88 (t, *J* = 7.6 Hz, 3H). ^13^C NMR (DMSO-*d*_6_): 174.06, 160.84, 159.79, 158.97, 157.02, 106.81, 95.41, 36.52, 22.25, 14.09. MS (ES-), *m/z*: 273 ([M-H]^−^, 100). Calculated for C_10_H_10_N_2_O_3_S: C, 50.41; H, 4.23; N, 11.76; Found: C, 49.92; H, 4.25; N, 11.43.

##### 5-Butyl-2-thioxo-2,3-dihydro-1*H*-pyrano[2,3-*d*]pyrimidine-4,7-dione **2-4**

Compound **2-4** was prepared according to the general procedure from thiobarbituric acid (1.44 g, 10 mmol) and ethyl 3-oxoheptanoate (2.24 mL, 12.6 mmol). Yield of compound **2-4**: 630 mg (25%) as light grey powder, Mp > 200 °C. ^1^H NMR (400 MHz, DMSO-*d*_6_): 12.64 (s, 1H), 5.90 (s, 1H), 2.83 (dd, *J* = 8.6, 6.4 Hz, 2H), 1.52-1.40 (m, 2H), 1.40-1.24 (m, 2H), 0.89 (t, *J* = 7.2 Hz, 3H). ^13^C NMR (DMSO-*d*_6_): 174.07, 161.18, 159.80, 158.94, 157.04, 106.72, 95.41, 33.37, 31.17, 22.36,14.18. MS (ES-), *m/z*: 251 ([M-H]^−^, 100). Calculated for C_11_H_12_ClN_2_O_3_S: C, 52.37; H, 4.79; N, 11.10; Found: C, 51.95; H, 4.57; N, 10.75.

##### 5-(4-Chlorobutyl)-2-thioxo-2,3-dihydro-1*H*-pyrano[2,3-*d*]pyrimidine-4,7-dione **2-6**

Compound **2-6** was prepared according to the general procedure from thiobarbituric acid (1.44 g, 10 mmol) and ethyl 7-chloro-3-oxoheptanoate (2.60 g, 12.6 mmol). Yield of compound **2-6**: 686 mg (24%) as white powder, Mp > 200 °C. ^1^H NMR (400 MHz, DMSO-*d*_6_): 12.65 (s, 1H), 5.93 (s, 1H), 3.64 (t, *J* = 6.6 Hz, 2H), 2.85 (t, *J* = 7.4 Hz, 2H), 1.75 (dt, *J* = 8.4, 6.6 Hz, 2H), 1.69-1.52 (m, 2H). ^13^C NMR (DMSO-*d*_6_): 174.12, 160.49, 159.90, 159.01, 157.04, 107.00, 96.39, 46.58, 32.76, 32.02, 26.34. MS (ES-), *m/z*: 285 ([M-H]^−^, 100). Calculated for C_11_H_11_ClN_2_O_3_S: C, 46.08; H, 3.87; N, 9.77; Found: C, 45.84; H, 3.77; N, 9.58.

##### General Procedure for the Synthesis of 2-(Substituted-thio)-5-alkyl-4H-pyrano]2,3-d]pyrimidine(1H)-diones **3-1**–**3-7**

A mixture of the appropriate 2-thioxo-pyranopyrimidine **2** (1 mmol, 1 eq) and piperidine (0.104 mL, 1.01 mmol) in ethanol (5 mL) was heated to reflux over ~10 min until the colour of the solution changed. The appropriate alkyl(aralkyl)bromide (1 mmol, 1 eq) was then added and the solution was refluxed for 10 min and stirred at rt for 24 h. After cooling, the precipitate was collected by filtration and washed with cold ethanol and water. The crude product was recrystallized from ethanol and dried under reduced pressure.

##### Ethyl 5-butyl-4,7-dioxo-1,7-dihydro-4*H*-(pyrano[2,3-*d*]pyrimidin-2-ylsulfanyl)acetate **3-1**

Compound **3-1** was prepared according to the general procedure from 2-thioxopyranopyrimidine **2-4** (252 mg, 1 mmol, 1 eq), piperidine (0.104 mL, 1.01 mmol), and ethyl 2-bromoacetate (0.11 mL, 1 mmol). Yield of compound **3-1**: 260 mg (78%) as light yellow powder. Mp: 156 °C. ^1^H NMR (400 MHz, DMSO-*d*_6_): 13.39 (s, 1H), 6.02 (s, 1H), 4.15 (q, *J* = 7.1 Hz, 2H), 4.08 (s, 2H), 2.93-2.79 (m, 2H), 1.47 (ddd, *J* = 11.9, 8.6, 6.0 Hz, 2H), 1.34 (h, *J* = 7.2 Hz, 2H), 1.21 (t, *J* = 7.2 Hz, 3H), 0.89 (t, *J* = 7.2 Hz, 3H). ^13^C NMR (DMSO-*d*_6_): 168.23, 165.05, 163.85, 160.28, 160.25, 159.10, 110.21, 99.26, 61.82, 33.51, 32.90, 31.09, 22.30, 14.41. MS (ES+), *m/z*: 339 ([M+H]^+^, 100). Calculated for C_15_H_18_N_2_O_5_S: C, 53.24; H, 5.36; N, 8.28; Found: C, 52.82; H, 5.27; N, 8.14.

##### [5-(4-Chlorobutyl)-4,7-dioxo-1,7-dihydro-4*H*-(pyrano[2,3-*d*]pyrimidin-2-ylsulfanyl)aceticacid ethyl ester **3-2**

Compound **3-2** was prepared according to the general procedure from 2-thioxopyranopyrimidine **2-6** (287 mg, 1 mmol, 1 eq), piperidine (0.104 mL, 1.01 mmol), and ethyl 2-bromoacetate (0.11 mL, 1 mmol). Yield of compound **3-2**: 310 mg (82%) as light yellow powder. Mp: 135-137 °C. ^1^H NMR (400 MHz, DMSO-*d*_6_): 13.42 (s, 1H), 6.06 (s, 1H), 4.15 (q, *J* = 7.1 Hz, 2H), 4.08 (s, 2H), 3.65 (t, *J* = 6.6 Hz, 2H), 2.91 (t, *J* = 7.4 Hz, 2H),1.76 (dt, *J* = 8.5 Hz, 2H), 1.69-1.56 (m, 2H), 1.21 (t, *J* = 7.1 Hz, 3H). ^13^C NMR (DMSO-*d*_6_): 182.21, 165.15, 163.95, 160.36, 159.66, 159.12, 110.56, 99.28, 61.87, 46.57, 32.95, 32.04, 26.28, 14.47. MS (ES+), *m/z*: 373 ([M+H]^+^, 100). Calculated for C_15_H_17_ClN_2_O_5_S: C, 48.32; H, 4.60; N, 7.51; Found: C, 47.97; H, 4.58; N, 7.41.

##### 2-[5-(4-Chlorobutyl)-4,7-dioxo-1,7-dihydro-4*H*-]pyrano[2,3-*d*]pyrimidin-2-ylsulfanyl]-acetic acid benzyl ester **3-3**

Compound **3-3** was prepared according to the general procedure from 2-thioxopyranopyrimidine **2-6** (287 mg, 1 mmol, 1 eq), piperidine (0.104 mL, 1.01 mmol), and benzyl 2-bromoacetate (0.158 mL, 1 mmol). Yield of compound **3-3**: 317 mg (73%) as light yellow powder, Mp: 153 °C. ^1^H NMR (400 MHz, DMSO-*d*_6_): 13.42 (s, 1H), 7.43–7.21 (m, 5H), 6.08 (s, 1H), 5.19 (s, 2H), 4.16 (s, 2H), 3.65 (t, *J* = 6.6 Hz, 2H), 2.91 (t, *J* = 7.4 Hz, 2H), 1.77 (dt, *J* = 8.6, 6.5 Hz, 2H), 1.64 (ddt, *J* = 12.9, 9.1, 6.0 Hz, 2H). ^13^C NMR (DMSO-*d*_6_): 168.21, 165.14, 163.90, 160.31, 159.65, 159.13, 136.20, 128.81, 128.45, 128.25, 110.60, 99.29, 67.07, 45.58, 32.96, 32.93, 32.06, 26.31. MS (ES+), *m/z*: 435 ([M+H]^+^, 100). Calculated for C_20_H_19_ClN_2_O_5_S: C, 55.24; H, 4.40; N, 6.44; Found: C, 55.14; H, 4.41; N, 6.39.

##### 2-[2-(4-Benzyloxy-phenyl)-1-methyl-2-oxo-ethylsulfanyl]-5-(4-chlorobutyl)-1*H*-pyrano[2,3-*d*]pyrimidine-4,7-dione **3-4**

Compound **3-4** was prepared according to the general procedure from 2-thioxopyranopyrimidine **2-6** (287 mg, 1 mmol, 1 eq), piperidine (0.104 mL, 1.01 mmol), and (4-benzyloxyphenyl) 2-bromopropanoate (319 mg, 1 mmol). Yield of compound **3-4**: 509 mg (97%) as light yellow powder, Mp: >200 °C. ^1^H NMR (400 MHz, DMSO-*d*_6_): 13.38 (s, 1H), 8.03 (d, *J* = 8.9 Hz, 2H), 7.50–7.29 (m, 5H), 7.17 (d, *J* = 8.9 Hz, 2H), 6.06 (s, 1H), 5.67 (q, *J* = 7.1 Hz, 1H), 5.22 (s, 2H), 3.64 (t, *J* = 6.6 Hz, 2H), 2.90 (dd, *J* = 8.4, 6.2 Hz, 2H), 1.82–1.70 (m, 2H), 1.68–1.59 (m, 2H), 1.57 (d, *J* =7.1 Hz, 3H). ^13^C NMR (DMSO-*d*_6_): 195.45, 165.23, 163.47, 163.30, 160.42, 159.68, 159.17, 136.82, 131.60, 128.99, 128.92, 128.54, 128.34, 128.21, 127.46, 115.51, 110.53, 99.43, 70.11, 45.72, 45.58, 32.96, 32.03, 26.29, 18.49. MS(ES-), *m*/*z*: 523 ([M-H]^−^, 100). Calculated for C_27_H_25_ClN_2_O_5_S: C, 61.77; H, 4.80; N, 5.34; Found: C, 61.67; H, 4.84; N, 5.24.

##### 2-[5-(4-Chlorobutyl)-4,7-dioxo-1,7-dihydro-4*H*-pyrano[2,3-*d*]pyrimidin-2-ylsulfanyl]-acetamide **3-5**

Compound **3-5** was prepared according to the general procedure from 2-thioxopyranopyrimidine **2-6** (287 mg, 1 mmol, 1 eq), piperidine (0.104 mL, 1.01 mmol), and iodoacetamide (184 mg, 1 mmol). Yield of compound **3-5**: 306 mg (89%) as light yellow powder, Mp: 168 °C. ^1^H NMR (400 MHz, DMSO-*d*_6_): 13.33 (s, 1H), 7.70 (s, 1H), 7.27 (s, 1H), 6.05 (s, 1H), 3.94 (s, 2H), 3.65 (t, *J* = 6.6 Hz, 2H), 2.91 (t, *J* = 7.4 Hz, 2H), 1.77 (dt, *J* = 15.1, 6.6 Hz, 2H), 1.63 (qd, *J* = 9.8, 8.8, 6.2 Hz, 2H). ^13^C NMR (DMSO-*d*_6_): 168.52, 165.30, 164.74, 160.48, 158.80, 159.22, 110.29, 99.17, 45.59, 34.83, 32.98, 32.05, 26.32. MS (ES+), *m*/*z*: 344 ([M+H]^+^, 100). Calculated for C_13_H_14_ClN_3_O_4_S: C, 45.42; H, 4.10; N, 12.22; Found: C, 45.33; H, 4.02; N, 11.90.

##### 2-2-[5-(4-Chlorobutyl)-4,7-dioxo-1,7-dihydro-4*H*-pyrano[2,3-*d*]pyrimidin-2-ylsulfanyl]-acetamino-cyclohex-1-enecarboxylic acid ethyl ester **3-6**

Compound **3-6** was prepared according to the general procedure from 2-thioxopyranopyrimidine 2-6 (287 mg, 1 mmol, 1 eq), piperidine (0.104 mL, 1.01 mmol), and ethyl 2-[(2-bromoacetyl)amino]-cyclohexene-1-carboxylate (290 mg, 1 mmol). Yield of compound **3-6**: 456 mg (92%) as light yellow powder, Mp: >200 °C. ^1^H NMR (400 MHz, DMSO-*d*_6_): 13.46 (s, 1H), 11.46 (s, 1H), 6.06 (s, 1H), 4.07 (m, 4H), 3.64 (m, 2H), 2.90 (m, 2H), 2.76 (bs, 2H), 2.25 (bs, 2H), 1.70–1.79 (m, 2H), 1.64 (m, 2H), 1.53 (m, 4H), 1.16 (t, *J* = 8.0 Hz, 3H). MS (ES-), *m*/*z*: 494 ([M-H]^−^, 100). Calculated for C_22_H_26_ClN_3_O_6_S: C, 53.28; H, 5.28; N, 8.47; Found: C, 53.03; H, 5.15; N, 8.28.

##### 2-2-[5-(4-Chlorobutyl)-4,7-dioxo-1,7-dihydro-4*H*-pyrano[2,3-*d*]pyrimidin-2-ylsulfanyl]-acetamino-benzoic acid **3-7**

Compound **3-7** was prepared according to the general procedure from 2-thioxopyranopyrimidine **2-6** (287 mg, 1 mmol, 1 eq), piperidine (0.104 mL, 1.01 mmol), and 2-(2-bromoacetylamino)benzoic acid (258 mg, 1 mmol). Yield of compound **3-7**: 310 mg (67%) as light yellow powder. Mp: 177-180 °C. ^1^H NMR (400 MHz, DMSO-*d*_6_): 13.40 (s, 1H), 11.40 (s, 1H), 7.38-7.17 (m, 4H), 6.01 (s, 1H), 3.65 (t, *J* = 6.5 Hz, 2H), 2.90 (t, *J* = 7.4 Hz, 2H), 2.89 (t, *J* = 7.4 Hz, 2H), 1.77 (dt, *J* = 8.4, 6.5 Hz, 2H), 1.64 (ddt, *J* = 12.9, 9.0, 6.0 Hz, 2H). MS (ES+), *m/z*: 465 ([M+H]^+^, 100). Calculated for C_20_H_18_ClN_3_O_6_S: C, 51.78; H, 3.91; N, 9.06; Found: C, 51.65; H, 3.89; N, 8.95.

### 3.3. Biological Tests

#### 3.3.1. Preparation of Cell Cultures and the Generation of Stable Cell Lines

Flp-In-293 cells (Invitrogen) were used for the generation of stable FFA3/GPR41 and FFA2/GRP43 cell lines using Flp-In System (Invitrogen). Flp-In-293 cells were cultured in Dulbecco’s modified Eagle medium (DMEM) supplemented with 10% fetal bovine serum (FBS), penicillin (100 U/mL), streptomycin (100 µg/mL), and zeocin (100 µg/mL). The cells were co-transfected with pOG44 (Invitrogen) and pcDNA5/FRT-FFA3 or pcDNA5/FRT-FFA2 using TurboFect (Thermo Fisher Scientific), and stable cell lines were generated according to the manufacturer’s protocol (Invitrogen). After transfection, the cells were cultured under selection pressure due to the presence of hygromycin B (100 µg/mL) in DMEM supplemented with 10% FBS, penicillin, and streptomycin.

Flp-In-293 cells stably expressing human HCA2/GPR109A receptor (laboratory collection) were grown in DMEM medium supplemented with 10% FBS, penicillin, streptomycin, and hygromycin B (100 µg/mL). All cells were cultured at 37 °C in a humidified incubator containing 5% CO_2_.

#### 3.3.2. THP-1 Cell Differentiation and Stimulation

The human THP-1 monocyte cell line was obtained from ATCC (American Type Culture Collection). The THP-1 cells were maintained in RPMI-1640 medium containing 10% FBS and antibiotics. The THP-1 monocytes were stimulated to differentiate into macrophages with phorbol 12-myristate 13-acetate (PMA) (50 ng/mL) for 24 h. The medium was exchanged for PMA-free medium for 48 h before the cells were used for experiments. For studying the effects of ligands on cytokine and receptor gene expression and cytokine secretion, the differentiated cells were stimulated for 4 h with 1 µg/mL LPS in the presence or absence of propionate (0.1 mM and 1 mM), niacin (50 µM), and the studied compounds (10 µM and 50 µM).

#### 3.3.3. Intracellular cAMP Inhibition Assay

For cAMP assay, the cells were distributed into a 384-well white microplate at a density of 12,000 cells per well and stimulated with the indicated compounds in the presence of 3 μM forskolin for 30 min. Compounds (50 µM) that were not active in the cAMP assay were tested for their antagonist activity in the presence of the respective receptor agonist: 5 µM niacin (HCA2/GPR109A), 1 µM **1-3** (FFA3/GPR41) or 1 µM **2-7** (FFA2/GRP43). The cells and the compounds were diluted in 1X phosphate-buffered saline (PBS) supplemented with 0.5 mM of 3-isobutyl-1-methylxanthine. The intracellular cAMP levels were measured using a Lance cAMP kit according to the manufacturer’s instructions. The plates were read on a PerkinElmer Victor 3V multilabel plate reader. The cAMP was quantitated in each sample by comparison to a calibration curve of known cAMP quantities provided in the kit.

#### 3.3.4. RNA Extraction and Quantitative Real-Time Polymerase Chain Reaction (qRT-PCR)

The total RNA was isolated from THP-1 cells using TRIzol reagent, according to the manufacturer’s protocol. First-strand cDNA was synthesized from 2 µg of RNA using oligo(dT) primers with RevertAid H Minus kit (Thermo Fisher Scientific, USA). The qRT-PCR procedure was performed using ABsolute Blue QPCR SYBR Green Master Mix reagent (Thermo Fisher Scientific, USA) with a ViiA7 real-time PCR detection system (Applied Biosystems, USA). The primer pairs that were employed for HCA2/GPR109A were forward 5′-TGATGCTCTTCATGTTGGCT-3′ and reverse 5′-GCTGAAGCTGCTGCACAA-3′; for FFA2/GPR43 were forward 5′-GCCTGGGTTATGTCCTTTGGTC-3′ and reverse 5′-CGGTGAAGTTCTCGTAGCAGGT-3′; for FFA3/GPR41 were forward 5′-TGCTGTTCCTGCCTTTCCGCAT-3′ and reverse 5′-GGAAGCGTTCAATGCTCACAGC-3′; for IL-6 were forward 5′-AGACAGCCACTCACCTCTTCAG-3′ and reverse 5′-TTCTGCCAGTGCCTCTTTGCTG-3′; for MCP-1 were forward 5′-AGAATCACCAGCAGCAAGTGTCC-3′ and reverse 5′-TCCTGAACCCACTTCTGCTTGG-3′; for TNF-α were forward 5′-CCCAGGGACCTCTCTCTAATCA-3′ and reverse 5′-AGCTGCCCCTCAGCTTGAG-3′; for RPS29 were forward 5′-CAAGATGGGTCACCAGCAG-3′ and reverse 5′- ATATTTCCGGATCAGACCGT-3′. The mRNA levels were quantified and normalized to the levels of the reference gene *RPS29* using the 2^−ΔΔCt^ method and presented as relative expression compared to the values for untreated cells.

#### 3.3.5. Quantification of Cytokines and Chemokines

Conditioned media were obtained from untreated and compound-treated THP-1 macrophages. The concentrations of the IL-6, TNF-α and MCP-1 were measured by Luminex Multiplex immunoassay (R&D Systems, USA) according to the manufacturer’s instructions, and analyzed on a Luminex 2000 (Luminex Corporation, Austin, TX, USA). Three independent repetitions in duplicate were made per sample. Concentrations were quantified with five parameters logistic (5-PL) curve fit and expressed in pg/mL.

#### 3.3.6. Statistical Analysis

The gene expression results are presented as the mean values ± SD for the indicated number of experiments. Student’s t-test was used to assess the statistical significance of differences. Significant differences were assumed for *p* < 0.05. The statistical and cAMP assay data analyses were performed using GraphPad Prism 5.0 software (GraphPad Software, San Diego, CA, USA).

#### 3.3.7. Characterisation of the Synthesized Compounds

One-dimensional ^1^H and ^13^C NMR spectra were recorded on a Varian 400 Mercury spectrometer at 400 MHz (for ^1^H nuclei) and 100 MHz (for ^13^C nuclei). The chemical shifts are presented in parts per million (ppm). Residual protons of deuterated solvents were used as internal standard for ^1^H NMR spectra (CDCl_3_: δ 7.26 ppm, DMSO-*d*_6_: δ 2.50). The deuterated solvent signals were used as internal standards for ^13^C NMR spectra.

Low-resolution mass spectra (MS) were recorded on a Waters ACQUITY UPLC system equipped with a BEH C_18_ column connected to a Waters SQ Detector 2 operating in the ESI positive ion mode. Elemental analyses were performed on an Elemental Combustion System ECS 4010 (Costech Instruments) at the Laboratory of Chromatography, Latvian Institute of Organic Synthesis. All target compounds had >95% purity. The purity of each compound was determined by HPLC on a Waters Alliance 2695 system equipped with an Altima C_18_ column, 5 µM, 4.6 × 150 mm and a Waters 2489 UV-VIS detector, using a gradient elution with acetonitrile/H_3_PO_4_ (0.1%) in water at a flow rate of 1 mL/min.

#### 3.3.8. The Evaluation of Redox Potentials

Cyclic voltammetry experiments were carried out using a PARSTAT 2273 electrochemical system. A stationary glassy carbon disk electrode (*d* = 0.8 mm) served as the working electrode, while the counter electrode was a Pt wire. The oxidation potentials were measured relative to an Ag/Ag^+^ reference electrode. Acetonitrile was dried over P_2_O_5_ and distilled; the distillate was stored over CaH_2_ and redistilled immediately prior to use. Recrystallized tetrabutylammonium tetrafluoroborate (TBATFB) was used as a supporting electrolyte at 0.1 M concentration.

#### 3.3.9. The Calculation of logP and TPSA

The logP (logP_OW_) and TPSA values were calculated with Chem3D Ultra 19 software (Perkin Elmer Informatics, Waltham, MA, USA). For the logP_OW_ calculations, a Molecular Networks module was used on molecular species, while salts were treated as cations.

## 4. Conclusions

In conclusion, our study has helped to characterise the similarity in the action of the test compounds on the short-chain fatty acid receptors FFA2/GPR43 and FFA3/GPR41, as well as to compare their activity towards the hydroxycarboxylic acid receptor HCA2/GPR109A. Hexahydroquinoline derivatives were mainly selective and potent on FFA3/GPR41 receptor in the low micromolar range, but also showed lower potency towards the FFA2/GPR43 and HCA2/GPR109A receptors.

The investigated oxo- and thioxopyranopyrimidines not only served as HCA2/GPR109A receptor ligands, but also stimulated the FFA3/GPR41 receptor in low micromolar or submicromolar ranges and the FFA2/GPR43 receptor, the latter with lower potency. The introduction of a chlorine atom in the alkyl chain led to different effects, diminishing the potency of the thioxo compounds **2-4** and **2-6** on FFA2/GPR43 receptor, but increasing the potency of the oxo compounds **2-5** and **2-7**. Lengthening the alkyl chain (*n*-propyl → *n*-butyl) increased the potency towards all of the studied receptors.

2-*S*-Alkyl derivatives of pyranopyrimidines showed a generally higher potency towards the FFA3/GPR41 receptor (EC_50_ = 0.38–8.2 µM) than towards the HCA2/GPR109A receptor. In the case of compounds **3-3** and **3-5**, the order of the potency levels was reversed, being higher towards HCA2/GPR109A.

Representatives from each group were able to modify the gene expression of all three receptors and to decrease LPS-mediated proinflammatory cytokine gene expression and secretion in macrophages, resembling the effect of the HCA2/GPR109A ligand niacin, as well as the FFA2/GPR43 and FFA3/GPR41 endogenous ligand propionate.

Hexahydroquinoline derivatives showed good electron-donating properties; these compounds could possess antioxidant activity. Other studied compounds also had appropriate calculated polar surface area, and they could have the ability to cross cell membranes and the blood-brain barrier.

Thus, our study has revealed several groups of compounds possessing selective activity towards one of the studied receptors, as well as groups of compounds possessing pleiotropic activity towards several receptors. The obtained data could be useful for further development of multitarget ligands.

## Figures and Tables

**Table 1:** The calculated physicochemical properties of oxo-hexahydroquinolines and their impact on the HCA2/GPR109A, FFA3/GPR41, and FFA2/GPR43 receptors- inhibition of forskolin-stimulated cAMP level.

No	Structural Formula	HCA2/ GPR109A, % of Inhibition ^b^	FFA3/ GPR41 EC_50,_ µM ^a^ or % of Inhibition ^b^; Emax,%^c^	FFA2/ GPR43 % of Inhibition ^b^	logP ^d^	TPSA ^d^	El.ox. pot., V ^e^	Ref. (for Synthesis)
**1-1**	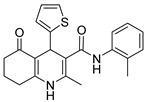	NA	1.79 ±0.3 ^a^ 96 ± 3 ^c^	NA	2.11 2.65 [22] ^d1^	58.20	1.19 1.64	[22]
**1-2**	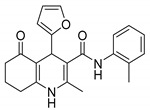	NA	0.61± 0.09 ^a^ 93 ± 5 ^c^	NA	1.69 2.18 [22] ^d1^	67.43	1.19 1.99	[21]
**1-3**	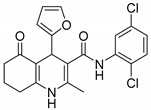	NA	0.32 ± 0.05 ^a^ 99 ± 4 ^c^	NA	2.71 2.90 [22] ^d1^	67.43		[21]
**1-4**	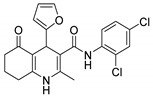	NA	0.23± 0.07 ^a^ 86 ± 7 ^c^	NA	2.71	67.43	1.24 1.67	M
**1-5**	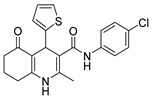	NA	45 ± 9 ^b^	NA	2.51	58.20		M
**1-6**	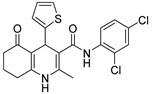	NA	1.4± 0.17 ^a^ 82 ± 7 ^c^	NA	3.13	58.20		M
**1-7**	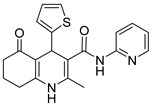	NA	NA	49 ± 8	1.35	70.56		M
**1-8**	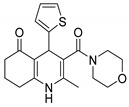	NA	28 ± 2 ^b^	NA	0.06	58.64		M
**1-9**	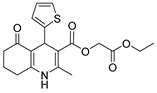	NA	1.9 ± 0.4 ^a^ 91 ± 6 ^c^	40 ± 2	0.97	81.70	1.18 1.68	M
**1-10**	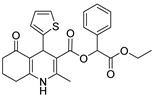	60 ± 5	49 ± 15 ^b^	31 ± 4	2.54	72.01	1.18 1.68	M
**1-11**	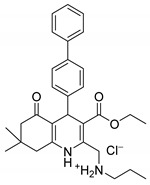	52 ± 5	51 ± 6 ^b^	55 ± 6	5.43	81.70		[33]
**1-12**	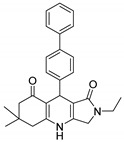	16 ± 4	16 ± 8 ^b^	21 ± 4	4.35	49.41		[33]
**1-13**	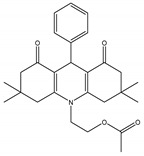	26 ± 8	30 ± 9 ^b^	32 ± 7	4.35	63.68		[34]
**1-14**	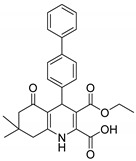	NA	NA	NA	4.70	92.70		[33]

NA—Not active up to the 50 µM concentration tested. M—Synthesis described in this publication. ^a^—EC_50_ value (µM). ^b^—Inhibition (%) of forskolin-stimulated intracellular accumulation of cAMP by 50 µM of the tested compound; forskolin-stimulated cAMP levels were set to 100%. ^c^—Maximal inhibition (%) of forskolin-stimulated cAMP. ^a; b; c^—Each value represents the mean ± SD calculated from at least three experiments, each performed in duplicate. ^d^—Calculated with Chem3D Ultra 19. ^d1^—Calculated with ChemDraw Professional Version 16. ^e^—Electrooxidation potential, V vs. Ag/Ag^+^.

**Table 2:**
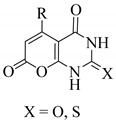
The calculated physicochemical properties of 2-oxo and 2-thioxopyranopyrimidines and their impact on HCA2/GPR109A, FFA3/GPR41, and FFA2/GPR43 inhibition of forskolin-stimulated cAMP level.

Compound	R	X	HCA2/ GPR109A EC_50_, µM ^a^; Emax,% ^c^	FFA3/ GPR41 EC_50_, µM ^a^, or % of Inhibition ^b^; Emax,% ^c^	FFA2/GPR43 EC_50_, µM ^a^, or % of Inhibition ^b^; Emax,% ^c^	LogP ^d^	TPSA ^d^	El.ox. pot., V ^e^	Ref. (for Synthesis)
**2-1**	CH_3_	S	7.10 ± 1.31 ^a^ 96 ± 5 ^c^	4.8 ± 0.36 ^a^ 89 ± 7 ^c^	19 ± 6.8 ^a^ 87 ± 8 ^c^	0.10	67.43		[36]
**2-2**	C_2_H_5_	S	0.49 ± 0.06 ^a^ 99 ± 2 ^c^	0.37 ± 0.08 ^a^9 6 ± 5 ^c^	0.92 ± 0.04 ^a^ 91 ± 4 ^c^	0.57	67.43		[19]
**2-3**	C_3_H_7_	S	0.87 ± 0.11 ^a^ 86 ± 3 ^c^	38 ± 2 ^b^	29 ± 3 ^b^	1.12	67.43		M
**2-4**	C_4_H_9_	S	0.55 ± 0.04 ^a^ 97 ± 4 ^c^	0.19 ± 0.03 ^a^ 95 ± 2 ^c^	0.74 ± 0.08 ^a^ 93 ± 6 ^c^	1.67	67.43		M
**2-5**	C_4_H_9_	O	0.051 ±0.007 ^a^ 93 ± 2 ^c^	28 ± 4 ^b^	NA	1.25	84.50	1.94	[18]
**2-6**	C_4_H_8_Cl	S	0.35 ± 0.02 ^a^ 94 ± 4 ^c^	0.34 ± 0.05 ^a^ 89 ± 3 ^c^	2.5 ± 0.4 ^a^ 83 ± 9 ^c^	1.36	67.43		M
**2-7**	C_4_H_8_Cl	O	0.054 ± 0.006 ^a^ 98 ± 3 ^c^	0.22 ± 0.03 ^a^ 89 ± 5 ^c^	0.38 ± 0.06 ^a^ 88 ± 7 ^c^	0.94	84.50	1.94	M

NA—Not active up to the 50 µM concentration tested. M—Synthesis described in this publication. ^a^—EC_50_ value (µM). ^b^—Inhibition (%) of forskolin-stimulated intracellular accumulation of cAMP by 50 µM of the tested compound; forskolin-stimulated cAMP levels were set to 100%. ^c^—Maximal inhibition (%) of forskolin-stimulated cAMP. ^a; b; c^—Each value represents the mean ± SD calculated from at least three experiments, each performed in duplicate. ^d^—Calculated with Chem3D Ultra 19. ^e^—Electrooxidation potential, V vs. Ag/Ag^+^.

**Table 3:**
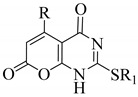
The electro-oxidation potentials and calculated physicochemical properties of S–alkylpyranopyrimidines and their impact on HCA2/GPR109A, FFA2/GPR43, and FFA3/GPR41 inhibition of forskolin- stimulated cAMP level.

Com-pound	R	R_1_	HCA2/ GPR109A EC_50,_ µM ^a^ or % of Inhibition ^b^; Emax, % ^c^	FFA3/ GPR41 EC_50,_ µM ^a^ or % of Inhibition ^b^; Emax, % ^c^	FFA2/ GPR43v% of Inhibition ^b^	logP ^d^	TPSA ^d^	El. ox.pot. V ^e^	Ref.
**3-1**	C_4_H_9_	CH_2_CO_2_C_2_H_5_	75 ± 11^a^ 93 ± 8^c^	1.6± 0.5^a^ 95 ± 3^c^	65 ± 1	1.81	97.74	1.97	M
**3-2**	C_4_H_8_Cl	CH_2_CO_2_C_2_H_5_	43 ±8^a^ 89 ± 4^c^	1.7± 0.2^a^ 91 ± 5^c^	64 ± 2	1.50	97.74		M
**3-3**	C_4_H_8_Cl	CH_2_CO_2_CH_2_Ph	17 ± 3^a^ 85 ± 10^c^	72 ± 2^b^	66 ± 6	2.79	97.74		M
**3-4**	C_4_H_8_Cl	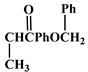	33 ± 16^b^	0.38± 0.1^a^ 95 ± 2^c^	49 ± 2	4.62	97.74	1.98	M
**3-5**	C_4_H_8_Cl	CH_2_CONH_2_	62± 10^a^ 87 ± 6^c^	20 ± 4^b^	42± 2	0.02	114.53	1.93	M
**3-6**	C_4_H_8_Cl	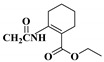	48 ± 8^b^	8.2 ± 1.3^a^ 89 ± 7^c^	62 ± 3	2.24	126.84		M
**3-7**	C_4_H_8_Cl	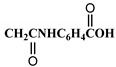	30 ± 6^b^	4.6 ± 0.6 ^a^ 87 ± 9^c^	52 ± 10	2.70	134.16		M

NA—Not active up to the 50 µM concentration tested. M—Synthesis described in this publication. ^a^—EC_50_ value (µM). ^b^—Inhibition (%) of forskolin-stimulated intracellular accumulation of cAMP by 50 µM of the tested compound; forskolin-stimulated cAMP levels were set to 100%. ^c^—Maximal inhibition (%) of forskolin-stimulated cAMP. ^a; b; c^—Each value represents the mean ± SD calculated from at least three experiments, each performed in duplicate. ^c^—Calculated with Chem3D Ultra 19. ^d^—Electrooxidation potential, V vs. Ag/Ag^+^.

## Data Availability

Methods of synthesis, analytical data of new compounds, and the results of biological activity characterisation are included within the article. NMR spectra of ^1^H and ^13^C are included within the Supplementary Information.

## Supplementary Materials

The following are available online at https://www.mdpi.com/article/10.3390/ph14100987/s1: ^1^H and ^13^C NMR spectra of compounds **1**, **2**, **3.**

## References

[1] Ramsay R.R., Popovic-Nicolic M.R., Nicolic K., Uliassi E., Bolognesi M.L. (2018). A perspective on multi-target drug discovery and design for complex diseases. Clin. Trans. Med..

[2] Hauser A.S., Chavali S., Masuho I., Jahn L.J., Martemyanov K.A., Gloriam D.E., M Babu M. (2018). Pharmacogenomics of GPCR Drug Targets. Cell.

[3] Brown A.J., M Goldsworthy S., Barnes A.A., Eilert M.M., Tcheang L., Daniels D., Muir A.I., Wigglesworth M.J., Kinghorn I., Fraser N.J. (2003). The orphan G protein-coupled receptors GPR41 and GPR43 are activated by propionate and other short chain carboxylic acids. J. Biol. Chem..

[4] Ahmed K. (2011). Biological roles and therapeutic potential of hydroxy-carboxylic acid receptors. Front. Endocrinol..

[5] Mishra S.P., Karunakar P., Taraphder S., Yadav H. (2020). Free fatty acid receptors 2 and 3 as microbial metabolite sensors to shape host health: Pharmacophysiological view. Biomedicines.

[6] Gille A., Bodor E.T., Ahmed K., Offermanns S. (2008). Nicotinic acid: Pharmacological effects and mechanisms of action. Ann. Rev. Pharmacol. Toxicol..

[7] Kimura I., Ozawa K., Inoue D., Imamura T., Kimura K., Maeda T., Terasawa K., Kashihara D., Hirano K., Tani T. (2013). The gut microbiota suppresses insulin-mediated fat accumulation via the short-chain fatty acid receptor GPR43. Nat. Commun..

[8] Miyamoto I., Hasegawa S., Kasubuchi M., Ichimura A., Nakajima A., Kimura I. (2016). Nutritional signalling via free fatty acid receptors. Int. J. Mol. Sci..

[9] Hoyles L., Snelling T., Umlai U., Nicholson J.K., Carding S.R., Glen R.C., McArthur S. (2018). Microbiome-host systems interactions: Protective effects of propionate upon the blood-brain barrier. Microbiome.

[10] Maslowski K.M., Vieira A.T., Ng A., Kranich J., Sierro F., Yu D., Schilter H.C., Rolp M.S., Mackay F., Artis D. (2009). Regulation of inflammatory responses by gut microbiota and chemoattractant receptor GPR43. Nature.

[11] Trompette A., Gollwitzer E.S., Yadava K., Sichelstiel A.K., Sprenger N., Ngom-Bru C., Blanchard C., Junt T., Nicod L.P., Harris N.L. (2014). Gut microbiota metabolism of dietary fiber influences allergic airway disease and hematopoiesis. Nat. Med..

[12] Li M., van Esch B.C.A.M., Wagenaar G.T.M., Garssen J., Folkerts G., Henricks P.A.J. (2018). Pro- and anti-inflammatory effects of short chain fatty acids on immune and endothelial cells. Eur. J. Pharmacol..

[13] Pongkorpsakol P., Moonwiriyakit A., Muanprasat C. (2017). Fatty acid and mineral receptors as drug targets for gastrointestinal disorders. Future Med. Chem..

[14] Carrette M.D., Quiroga J., López R., Hidalgo M.A., Burgos R.A. (2021). Participation of short-chain fatty acids and their receptors in gut inflammation and colon cancer. Front. Physiol..

[15] Kimura I., Ichimura A., Ohue-Kitano R., Igarashi M. (2020). Free fatty acid receptors in health and disease. Phys. Rev..

[16] Hauser A., Atwood M.M., Back-Andersen M., Schiöth H.B., Gloriam D.E. (2017). Trends in GPCR drug discovery: New agents, targets and indications. Nat. Rev. Drug Discov..

[17] Grundmann M., Bender E., Schamberger J., Eitner F. (2021). Pharmacology of free fatty acid receptors and their allosteric modulators. Int. J. Mol. Sci..

[18] Palani A., Rao A., Chen X., Huang X., Su J., Tang H., Huang Y., Qin J., Xiao D., Degrado S. (2012). Discovery of SCH 900271, a potent nicotinic acid receptor agonist for the treatment of dyslipidemia. ACS Med. Chem. Lett..

[19] Huang X., Su J., Rao A.U., Tang H., Zhou W., Zhu X., Chen X., Liu Z., Huang Y., Degrado S. (2012). SAR studies of C2 ethers of 2H-pyrano[2,3-d]pyrimidine-2,4,7(1H,3H)triones as nicotinic acid receptor (NAR) agonists. Bioorg. Med. Chem. Lett..

[20] Leonard J.N., Chu Z.L., Bruce M.A., Boatman P.D. (2016). GPR41 and modulators thereof for the treatment of insulin-related disorders. U.S. Patent.

[21] Hudson B.D., Christiansen E., Murdoch H., Jenkins L., Hansen A.H., Madsen O., Ulven T., Milligan G. (2014). Complex pharmacology of novel allosteric free fatty acid 3 receptor ligands. Mol. Pharm..

[22] Ulven E.R., Quon T., Sergeev E., Barki N., Brvar M., Hudson B.D., Dutta P.A.H., Bielefeldt L.Ø., Tobin A.B. (2020). Structure-activity relationship studies of tetrahydroquinolone free fatty acid receptor 3 modulators. J. Med. Chem..

[23] Anighoro A., Bajorath J., Rastelli G. (2014). Polypharmacology: Challenges and opportunities in drug discovery. J. Med. Chem..

[24] Ma H., Boshi H., Zhang Y. (2020). Recent advances in multitarget-directed ligands targeting G-protein-coupled receptors. Drug Discov. Today.

[25] De Silva M.F., Dias K.S.T., Gontijo V.S., Ortiz C.J.C., Viegas C. (2018). Multi-target directed drugs as a modern approach for drug design towards Alzheimer’s disease: An update. Curr. Med. Chem..

[26] Wright J.W., Harding J.W. (2013). The development of multi-target-directed ligands (MTDL) to treat Alzheimer’s disease. Front. Clin. Drug Res. Alzheimer Disord..

[27] Perone R., Albertini C., Uliassi E., Di Pietri F., de Pinheiro P.S.M., Petralla S., Rizzardi N., Fato R., Pulkrabkova L., Soukup O. (2021). Turning Donepezil into a multi-target directed ligand through a merging strategy. ChemMedChem.

[28] Ramalakshmi N., Remya R.S., Nalini C.N. (2021). Multitarget directed ligand approaches for Alzheimer’s disease: A Comprehensive Review. Mini-Rev. Med. Chem..

[29] Rossi M., Freschi M., de Camargo Nascente L., Salerno A., de Melo Viana Teixeira S., Nachon F., Chantegreil F., Soukup O., Prchal L., Malaguti M. (2021). Sustainable drug discovery of multi-target directed ligands for Alzheimer’s disease. J. Med. Chem..

[30] Digby J.E., Martinez F., Jefferson A., Ruparelia N., Chai J., Wamil M., Greaves D.R., Choudhury R.P. (2012). Anti-inflammatory effects of nicotinic acid in human monocytes are mediated by GPR109A dependent mechanisms. Arterioscler. Thromb. Vasc. Biol..

[31] Ang Z., Er J., Tan N., Lu J., Liou Y.-C., Grosse J., Ding J.L. (2016). Human and mouse monocytes display distinct signalling and cytokine profiles upon stimulation with FFAR2/FFAR3 short-chain fatty acid receptor agonists. Sci. Rep..

[32] Benfeito S., Oliveira C., Fernandes C., Cagide F., Teixeira J., Amorim R., Garrido J., Martins C., Sarmento B., Silva R. (2019). Fine-tuning the neuroprotective and blood-brain barrier permeability profile of multi-target agents designed to prevent progressive mitochondrial dysfunction. Eur. J. Med. Chem..

[33] Langle D., Marquardt V., Heider E., Vigante B., Duburs G., Luntena I., Flötgen D., Golz C., Strohmann C., Koch O. (2015). Design, synthesis and 3D-QSAR studies of novel 1,4-dihydropyridines as TGFb/Smad inhibitors. Eur. J. Med. Chem..

[34] Stankevich E., Vanags G. (1961). Polynuclear heterocyclic compounds.VII. Reaction of bis-dimedonyl methanes with compounds containing the amino group. Reduction of octahydroacridinediones. Latvijas PSR Zinātņu Akadēmijas Vēstis.

[35] Kin J., Rao A., Chen X., Zhu X., Liu Z., Huang X., Degrado S., Huang Y., Xiao D., Aslanian R. (2011). Discovery of a potent nicotinic acid receptor agonist for the treatment of dyslipidemia. ACS Med. Chem. Lett..

[36] Ridi M., Feroci G. (1950). Barbituric acid and its derivatives. VII Some reaction with ethyl acetate. Gazz. Chim. Ital..

[37] Vijesh A.M., Isloor A.M., Peethambar S.K., Shivananda K.N., Arulmoli T., Isloor N.A. (2011). Hantzsch reaction: Synthesis and characterization of some new 1,4-dihydropyridine derivatives as potent antimicrobial and antioxidant agents. Eur. J. Med. Chem..

[38] Augustyniak A., Bartosz G., Čipak A., Duburs G., Horáková L., Luczaj W., Majekova M., Odysseos A.D., Rackova L., Skrzydlewska E. (2010). Natural and synthetic antioxidants: An updated overview. Free Radic. Res..

[39] Velena A., Zarkovic N., Gall Troselj K., Bisenieks E., Krauze A., Poikans J., Duburs G. (2016). 1,4-Dihydropyridine derivatives: Dihydronicotinamide analogues—model compounds targeting oxidative stress. Oxid. Med. Cell Longev..

[40] Da Costa Cabrera D., Santa-Helena E., Leal H.P., de Moura R.R., Nery L.E.M., Neves Gonçalves C.A., Russowsky D., D’Oca M.G.M. (2019). Synthesis and antioxidant activity of new lipophilic dihydropyridines. Bioorg. Chem..

[41] Rojstaczer N., Triggle D.J. (1996). Structure-function relationships of calcium antagonists. Effect on oxidative modification of lower density lipoprotein. Biochem. Pharmacol..

[42] Rosenkranz A.C., Lob H., Breitenboch T., Berkels R., Roesen R. (2006). Endothelial antioxidant actions of dihydropyridines and angiotensin coverting enzyme inhibitors. Eur. J. Pharmacol..

[43] Malek R., Maj M., Wnorowski A., Jóźwiak K., Martin H., Iriepa I., Moraleda I., Chabchoub F., Marco-Contelles J., Ismaili L. (2019). Multi-target 1,4-dihydropyridines showing calcium channel blockade and antioxidant capacity for Alzheimer’s disease therapy. Bioorg. Chem..

[44] Milkovic L., Vukovic T., Zarkovic N., Tatzber F., Bisenieks E., Kalme Z., Bruvere I., Ogle Z., Poikans J., Velena A. (2018). Antioxidative 1,4-dihydropyridine derivatives modulate oxidative stress and growth of human osteoblast-like cells in vitro. Antioxidants.

[45] Sürücü O., Bolat G., El-Khouly A., Gözde Gündüz M., Simşek R., Abacı S., Kuralay F., Şafak C. (2016). Electrochemical detection of antioxidant activity of 1,4-dihydropyridine derivatives. Hacet. J. Biol. Chem..

[46] Macha R., Ravindrachary K., Jayasree G.P.L., Tigulla P. (2017). Spectrophotometric antioxidant bioassay and molecular modelling studies of ethyl 4-substituted-1,4,5,6,7,8-hexahydro-2,7,7-trimethyl-5-oxoquinoline-3-carboxylate derivatives. IJPSR.

[47] Brinkerhoff R.C., Santa-Helena E., do Amaral P.C., da Cabrera D., Ongaratto R.F., de Oliveira P.M., da D’Oca C.M., Gonçalves C.A.N., Nery L.E.M., D’Oca M.G.M. (2009). Evaluation of the antioxidant activities of fatty polyhydroquinolines synthesized by Hantzsch multicomponent reactions. RSC Adv..

[48] Tavakkoli Z., Goljani H., Gunduz M.G., Tahir M.N., Nematolllahi D. (2020). Electrochemical studies of newly synthesized 1,4-dihydropyridine-based hexahydroquinoline derivatives. J. Electrochem. Soc..

[49] Lipinski C.A. (2000). Drug-like properties and the causes of poor solubility and poor permeability. J. Pharmacol. Toxicol. Methods.

[50] Zhong H.A., Mashinson V., Woolman T.A., Zha M. (2013). Understanding the molecular properties and metabolism of top prescribed drugs. Curr. Top. Med. Chem..

[51] Wakade C., Chong R., Bradley E., Thomas B., Morgan J. (1098). Upregulation of GPR109A in Parkinson’s Disease. PLoS ONE.

[52] Falomir-Lockhart L.J., Cavazzutti G.F., Gimenez E., Toscani A.M. (2019). Fatty Acid Signaling Mechanism in Neural Cells: Fatty Acid Receptors. Front. Cell. Neurosci..

[53] Shityakov S., Neuhaus W., Dandekar T., Förster C. (2013). Analysing molecular polar surface descriptors to predict blood-brain barrier permeation. Int. J. Comput. Biol. Drug Des..

[54] Cox M.A., Jackson J., Stanton M., Rojas-Triana A., Bober L., Laverty M., Yang X., Zhu F., Liu J., Wang S. (2009). Short-chain fatty acids act as antiinflammatory mediators by regulating prostaglandin E(2) and cytokines. World. J. Gastroenterol..

[55] Tsalamandris S., Antonopoulos A.S., Oikonomou E., Papamikroulis G.A., Vogiatzi G., Papaioannou S., Deftereos S., Tousoulis D. (2019). The role of inflammation in Diabetes: Current concepts and future perspectives. Eur. Cardiol..

[56] Priyadarshini M., Lednovich K., Xu K., Gough S., Wicksteed B., Layden B.T. (2021). FFAR from the gut microbiome crowd: SCFA receptors in T1D pathology. Metabolites.

