# Supplementary material for: The Specificity and Broad Multitarget Properties of Ligands for the Free Fatty Acid Receptors FFA3/GPR41 and FFA2/GPR43 and the Related Hydroxycarboxylic Acid Receptor HCA2/GPR109A
Source: Pharmaceuticals (Basel). 2021 Sep 28;14(10):987. doi: 10.3390/ph14100987 (PMC8537386; doi:10.3390/ph14100987)
Supplement: Supplementary file 1 [file pharmaceuticals-14-00987-s001.zip › pharmaceuticals-1343049-supplementary.pdf]

Supplementary Materials

Pharmaceuticals

The specificity and broad multitarget properties of ligands for the free fatty acid receptors FFA3/GPR41 and FFA2/GPR43 and the related hydroxycarboxylic acid receptor HCA2/GPR109A

Egils Bisenieks[1], Brigita Vigante[1], Ramona Petrovska[2], Baiba Turovska[1], Ruslan Muhamadejev[1], Vitalijs Soloduns[1], Astrida Velena[1], Karlis Pajuste[1], Luciano Saso[3], Janis Klovins[2], Gunars Duburs[1], Ilona Mandrika[2]

[1]Latvian Institute of Organic Synthesis, Aizkraukles iela 21, Riga LV-1006, Latvia
[2]Latvian Biomedical Research and Study Centre, Ratsupites iela 1, Riga LV-1067, Latvia
[3]Department of Physiology and Pharmacology "Vittorio Erspamer", Sapienza University, P.le Aldo Moro 5, 00185, Rome, Italy

Examples of ^{1}H and ^{13}C NMR spectra of compounds **1**, **2**, **3** and **S2-S25**

MAS-2-VS-21-B-2-X.12.fid

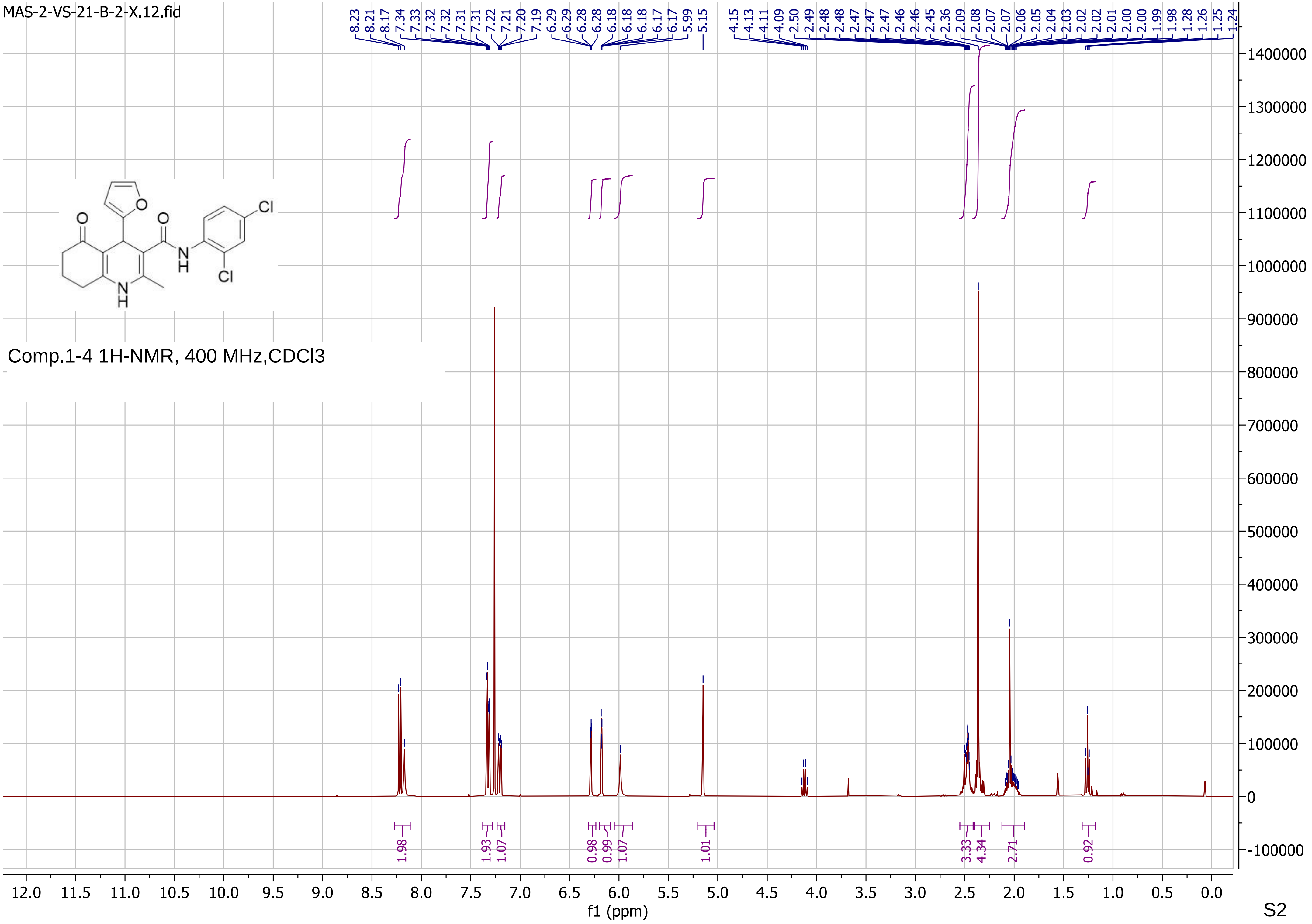

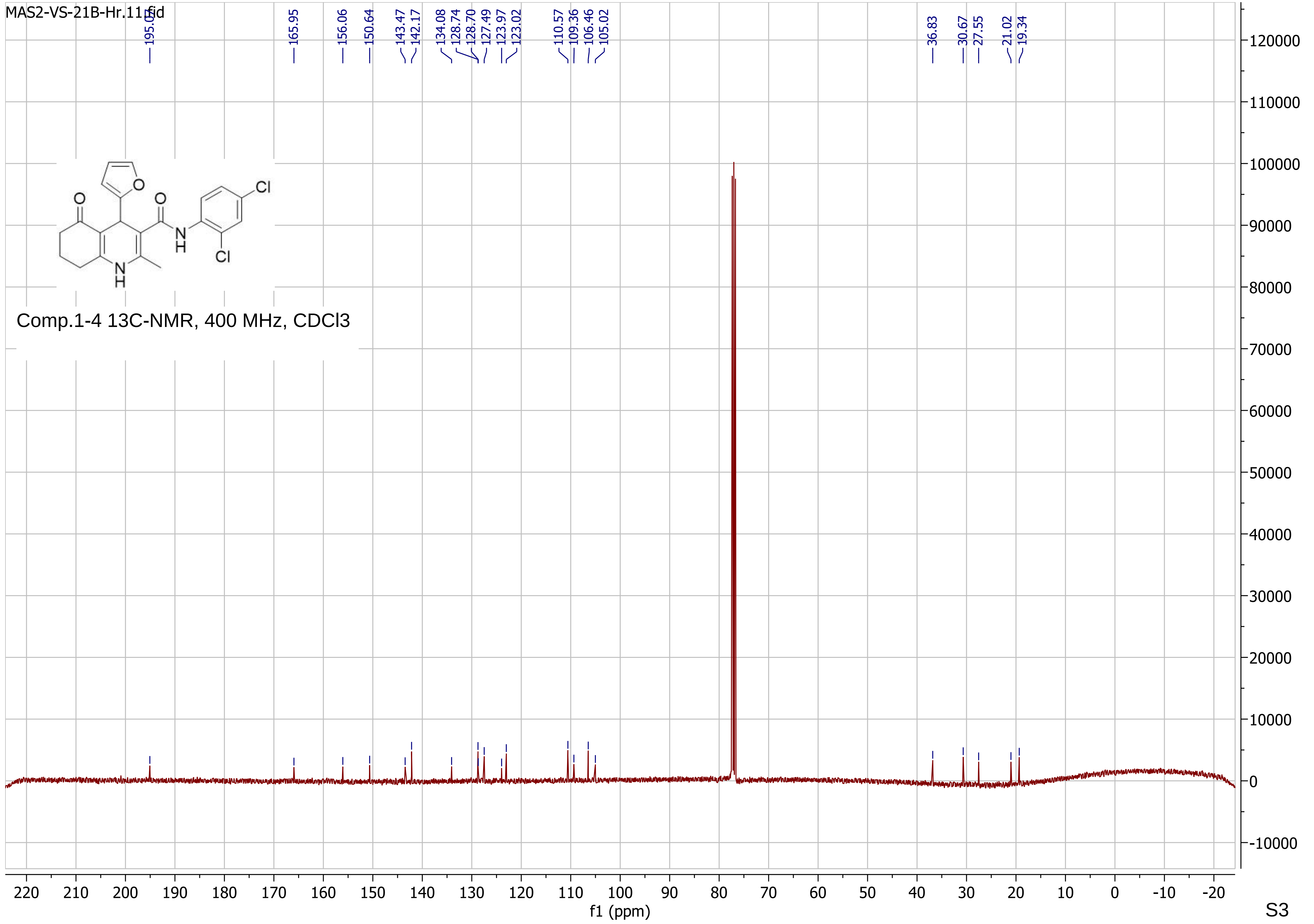
MAS2-VS-21B-Hr.11.fid
195.0[illegible]
165.95
156.06
150.64
143.47
142.17
134.08
128.74
128.70
127.49
123.97
123.02
110.57
109.36
106.46
105.02
36.83
30.67
27.55
21.02
19.34
O
O
O
N
H
Cl
Cl
N
H
Comp.1-4 13C-NMR, 400 MHz, CDCl3
f1 (ppm)

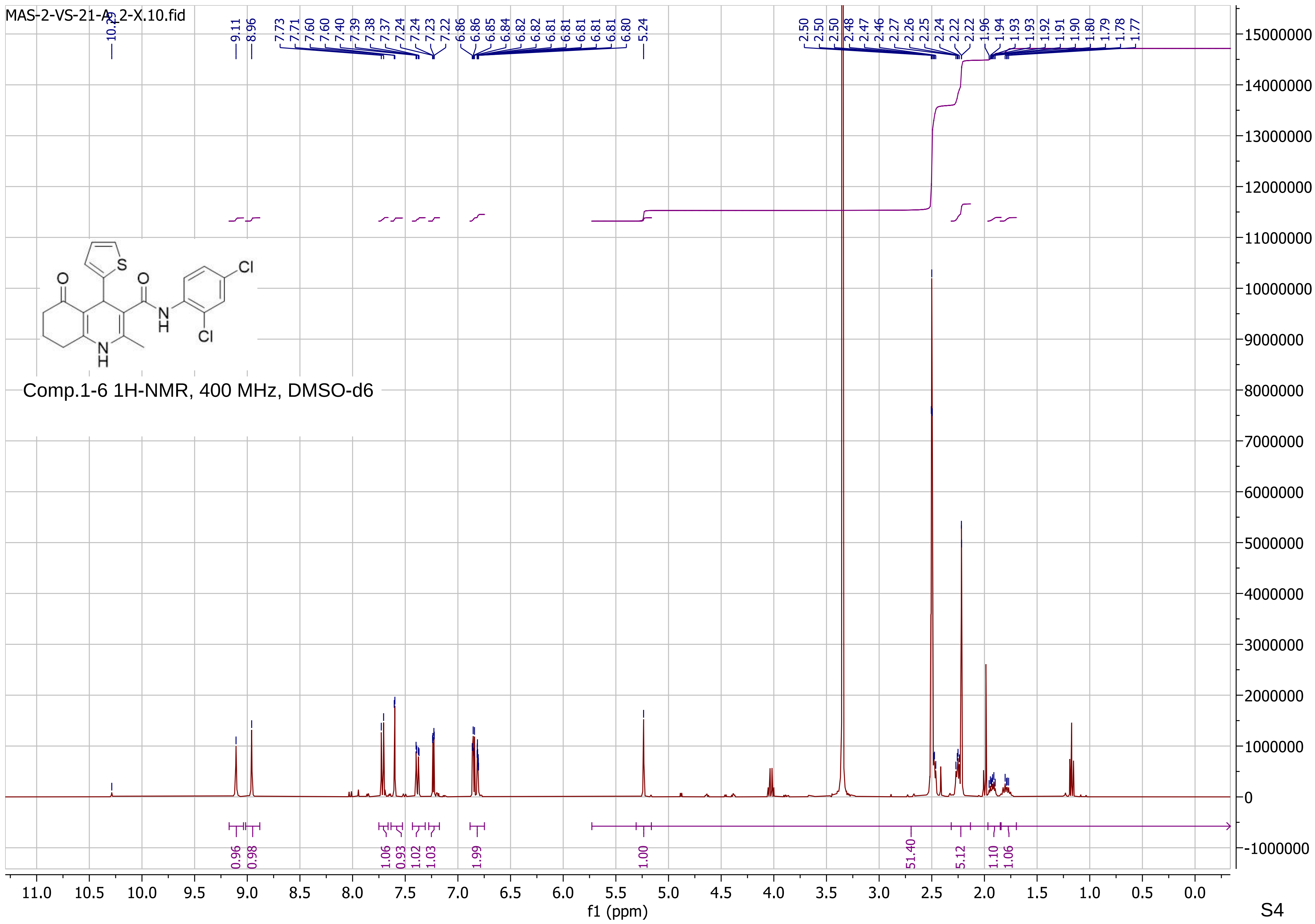
MAS-2-VS-21-A_2-X.10.fid
Comp.1-6 1H-NMR, 400 MHz, DMSO-d6
f1 (ppm)

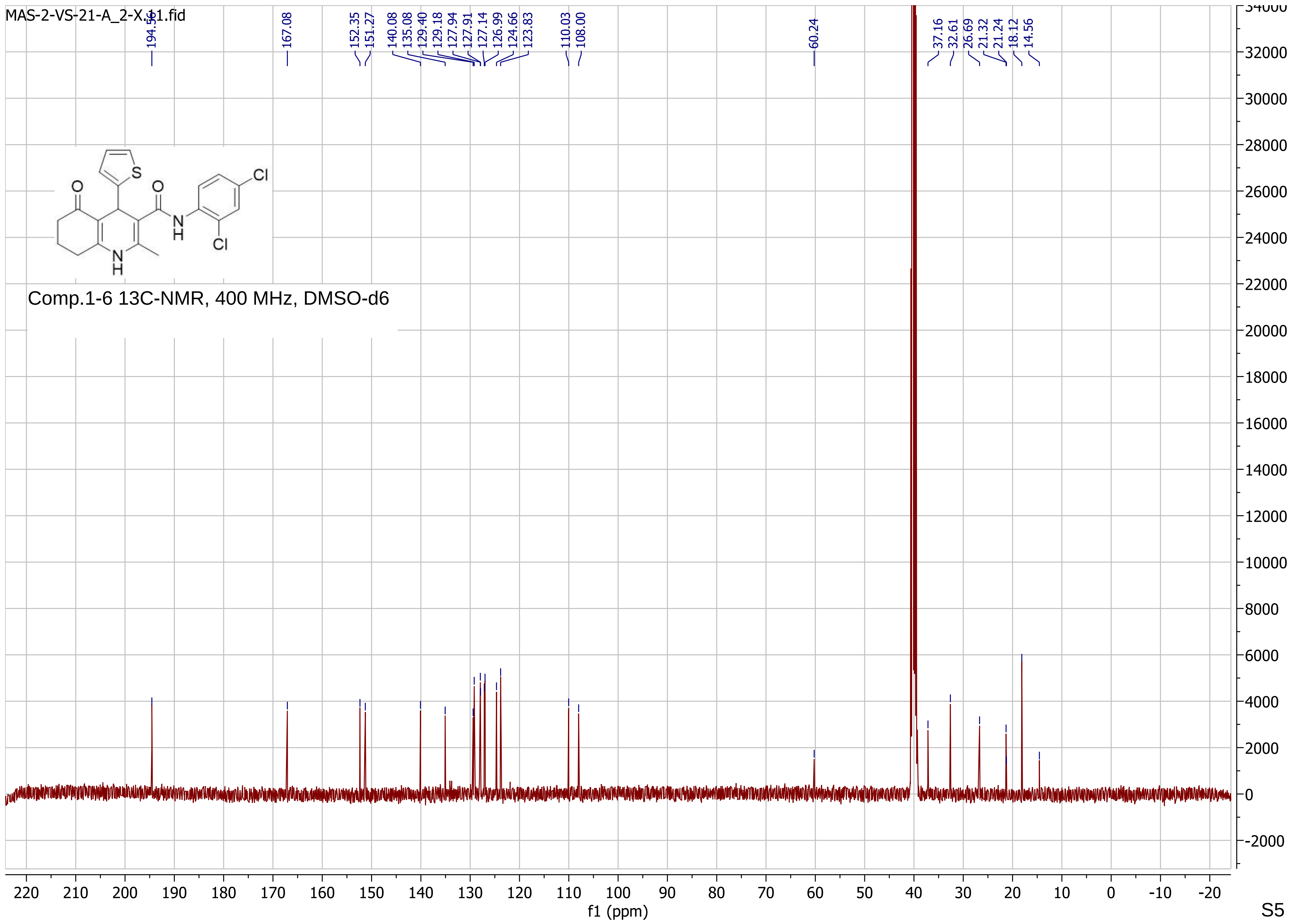
MAS-2-VS-21-A_2-X.s1.fid
194.56
167.08
152.35
151.27
140.08
135.08
129.40
129.18
127.94
127.91
127.14
126.99
124.66
123.83
110.03
108.00
60.24
37.16
32.61
26.69
21.32
21.24
18.12
14.56
O
S
O
Cl
N
H
N
H
Cl
Comp.1-6 13C-NMR, 400 MHz, DMSO-d6
f1 (ppm)

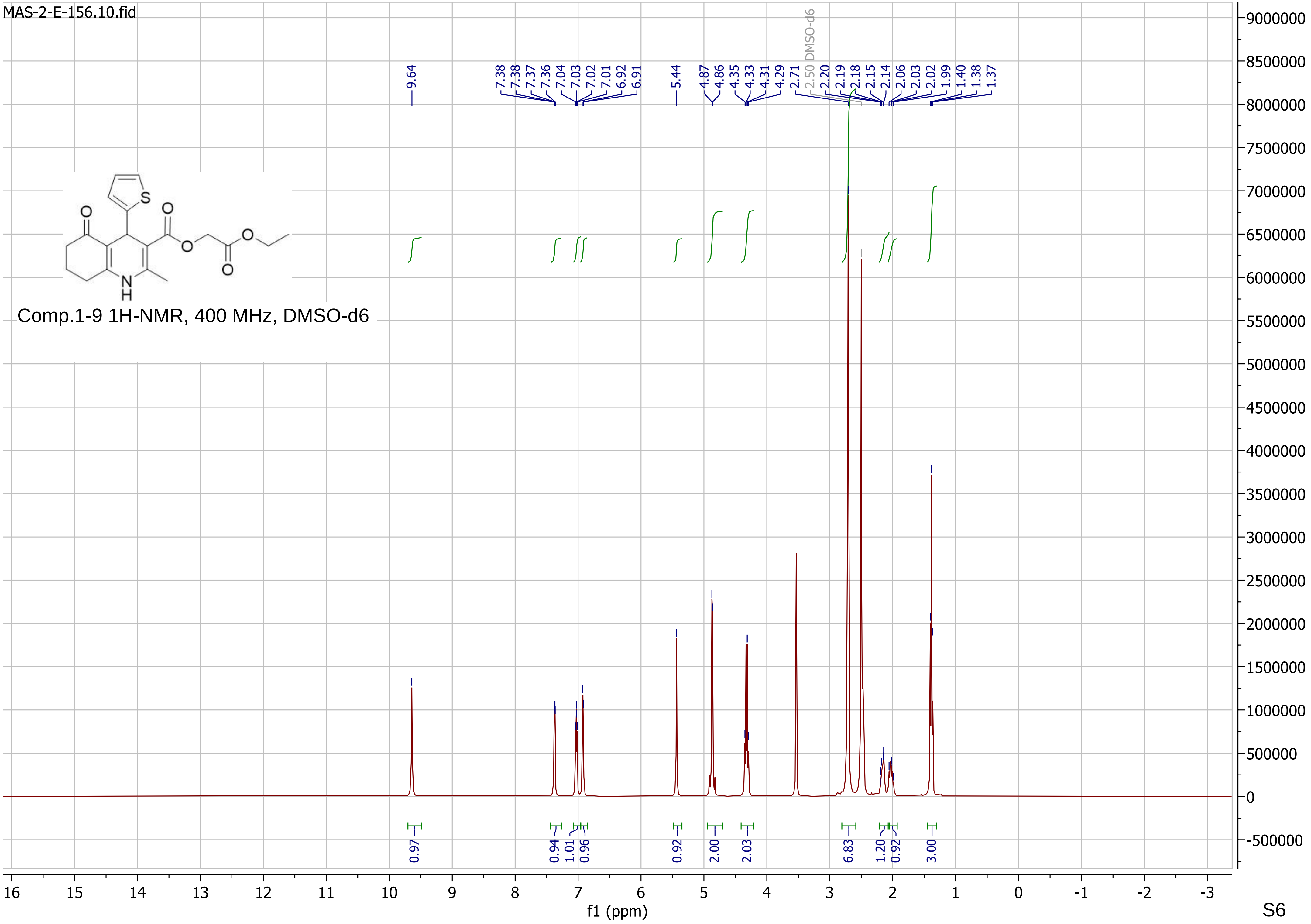
MAS-2-E-156.10.fid
Comp.1-9 1H-NMR, 400 MHz, DMSO-d6
9.64
7.38 7.38 7.37 7.36 7.04 7.03 7.02 7.01 6.92 6.91
5.44
4.87 4.86 4.35 4.33 4.31 4.29
2.71
2.50 DMSO-d6
2.20 2.19 2.18 2.15 2.14 2.06 2.03 2.02 1.99 1.40 1.38 1.37
0.97 0.94 1.01 0.96 0.92 2.00 2.03 6.83 1.20 0.92 3.00
f1 (ppm)

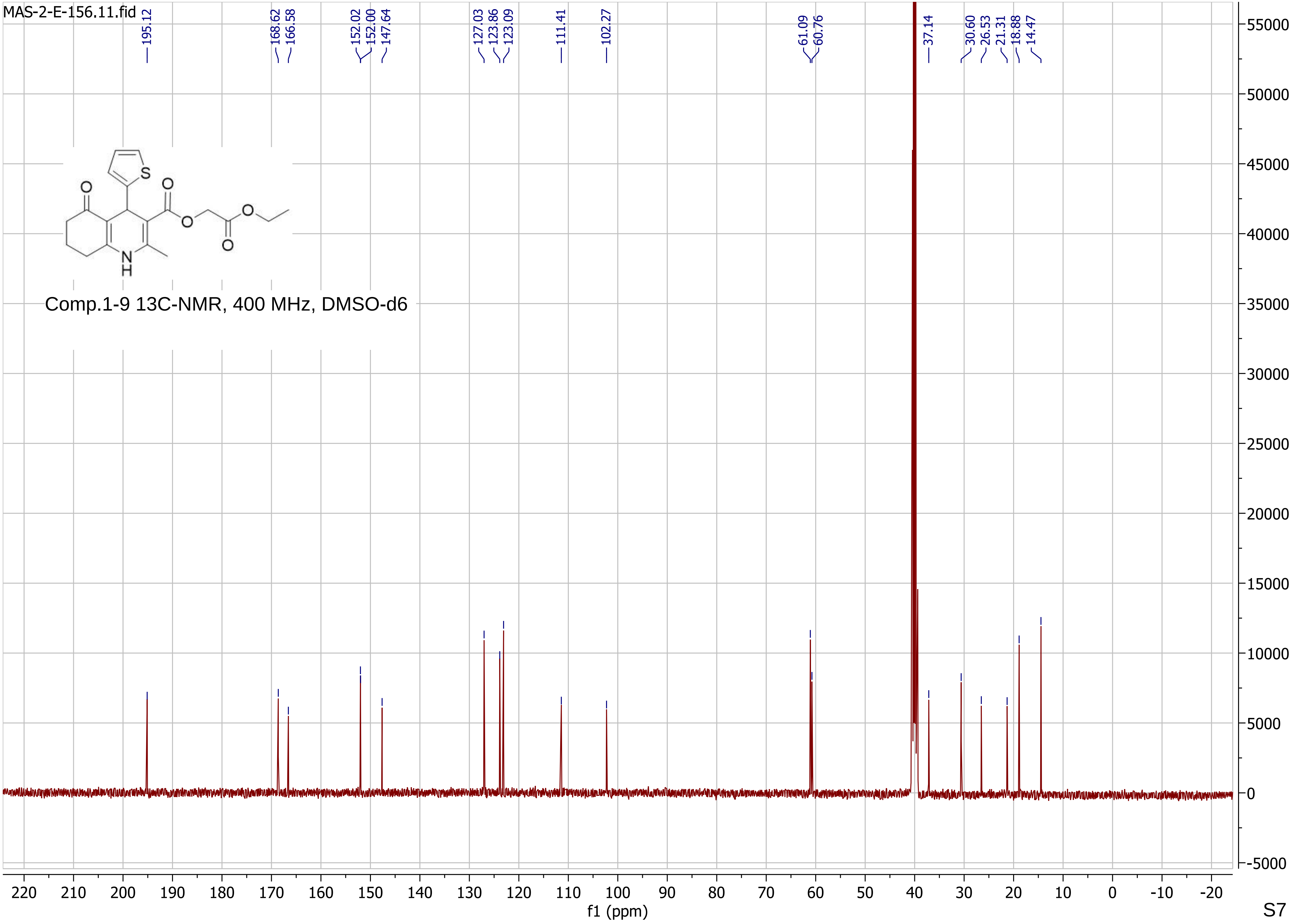

Comp.1-9 13C-NMR, 400 MHz, DMSO-d6

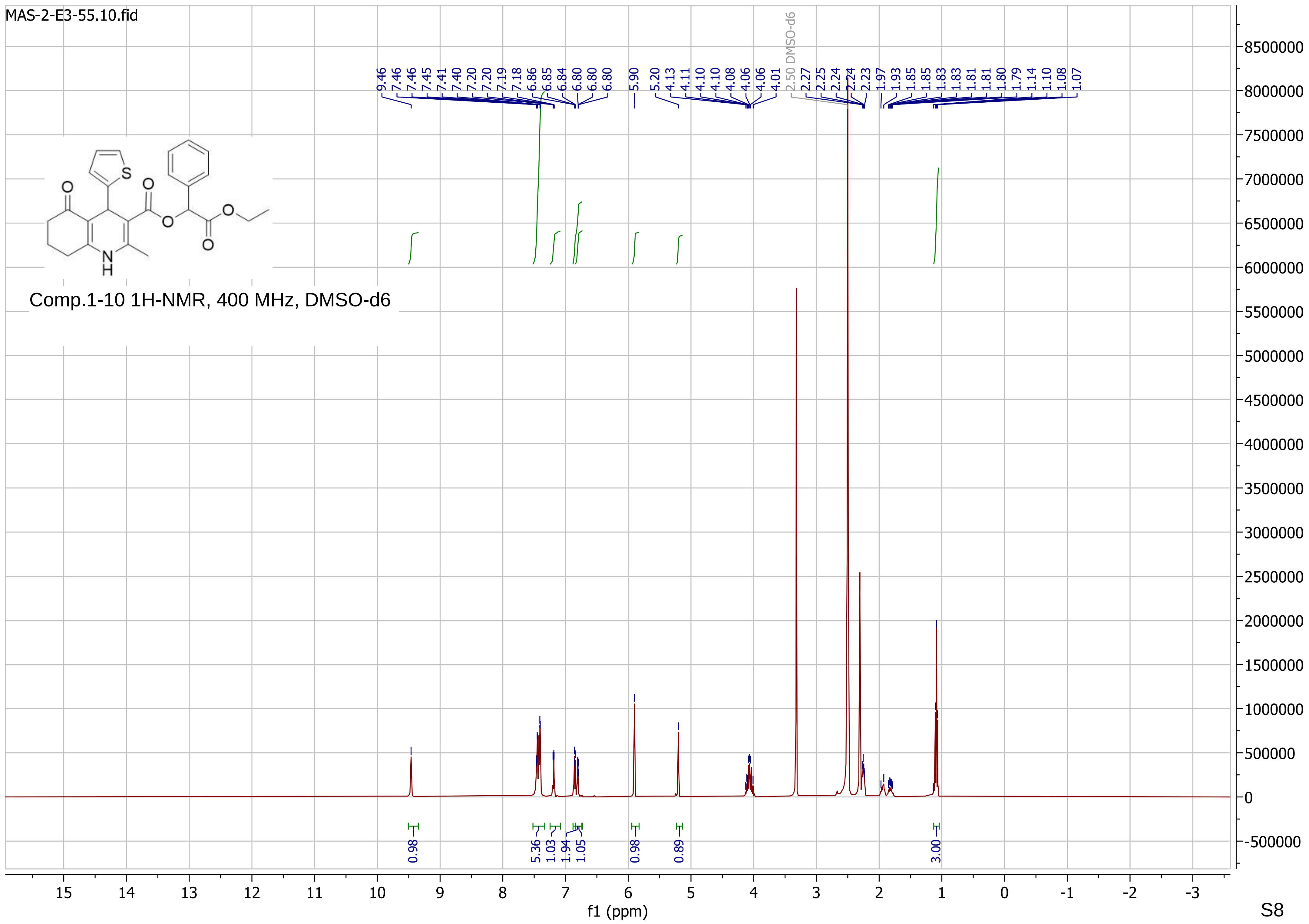
MAS-2-E3-55.10.fid
Comp.1-10 1H-NMR, 400 MHz, DMSO-d6
9.46
7.46
7.46
7.45
7.41
7.40
7.20
7.20
7.19
7.18
6.86
6.85
6.84
6.80
6.80
6.80
5.90
5.20
4.13
4.11
4.10
4.10
4.08
4.06
4.06
4.01
2.50 DMSO-d6
2.27
2.25
2.24
2.24
2.23
1.97
1.93
1.85
1.85
1.83
1.83
1.81
1.81
1.80
1.79
1.14
1.10
1.08
1.07
0.98
5.36
1.03
1.94
1.05
0.98
0.89
3.00
f1 (ppm)

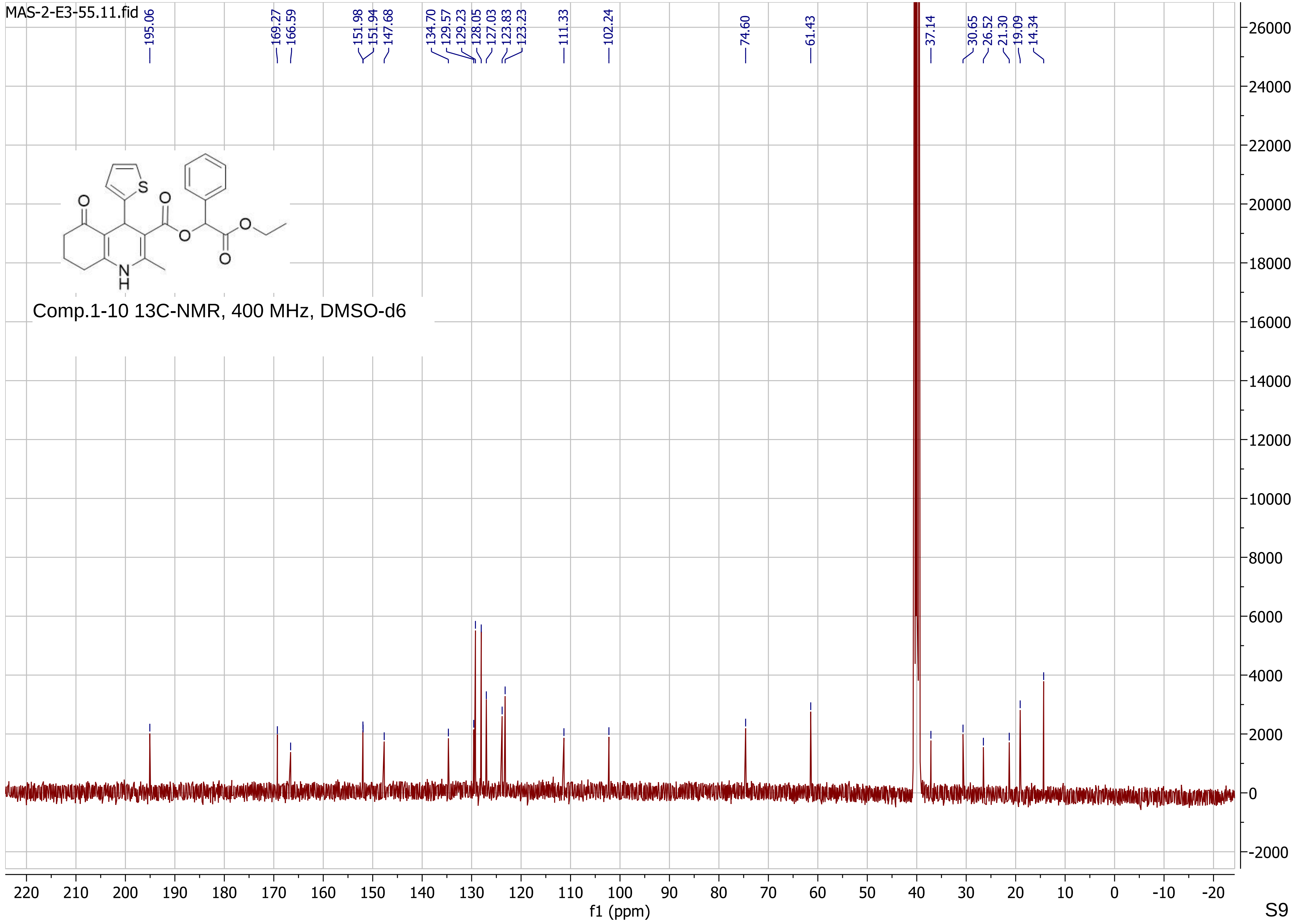
MAS-2-E3-55.11.fid
195.06
169.27
166.59
151.98
151.94
147.68
134.70
129.57
129.23
128.05
127.03
123.83
123.23
111.33
102.24
74.60
61.43
37.14
30.65
26.52
21.30
19.09
14.34
Comp.1-10 13C-NMR, 400 MHz, DMSO-d6
f1 (ppm)

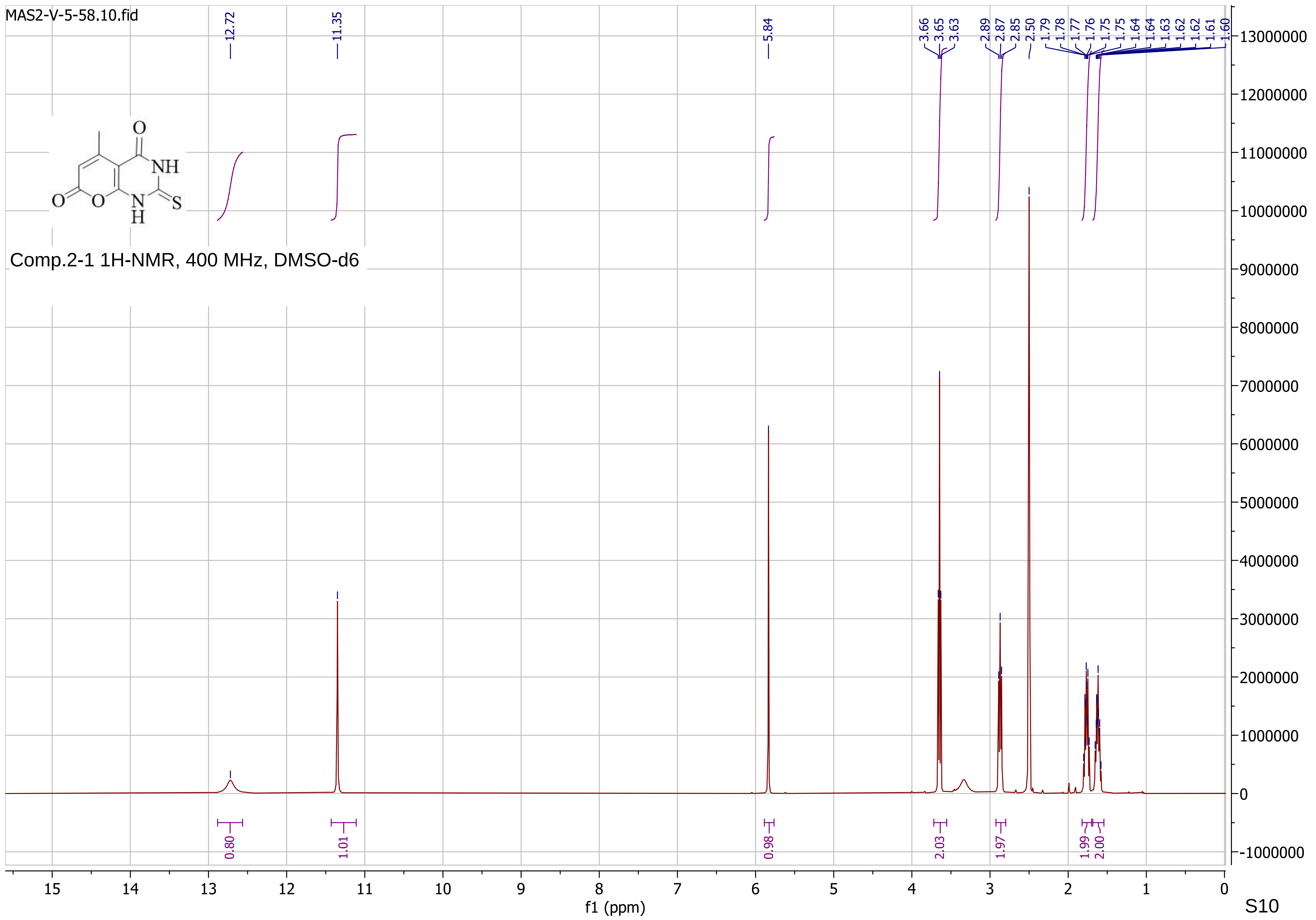
MAS2-V-5-58.10.fid
12.72
11.35
5.84
3.66
3.65
3.63
2.89
2.87
2.85
2.50
1.79
1.78
1.77
1.76
1.75
1.75
1.64
1.64
1.63
1.62
1.62
1.61
1.60
O
NH
S
N
H
O
O
Comp.2-1 1H-NMR, 400 MHz, DMSO-d6
0.80
1.01
0.98
2.03
1.97
1.99
2.00
f1 (ppm)

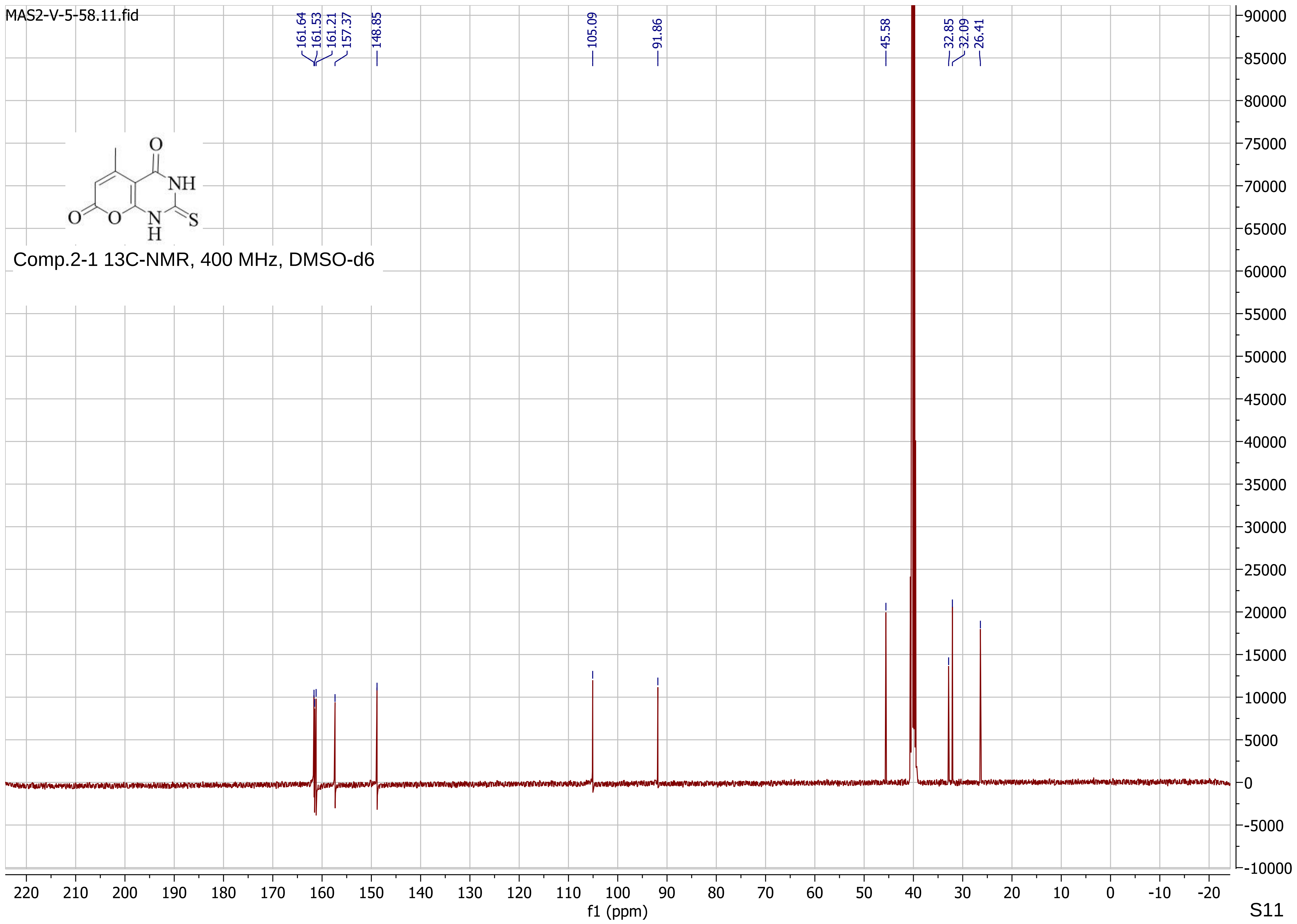
MAS2-V-5-58.11.fid
161.64
161.53
161.21
157.37
148.85
105.09
91.86
45.58
32.85
32.09
26.41
O
NH
S
N
H
O
O
Comp.2-1 13C-NMR, 400 MHz, DMSO-d6
f1 (ppm)

MAS2-V-3-60.10.fid

Comp.2-2 1H-NMR, 400 MHz, DMSO-d6

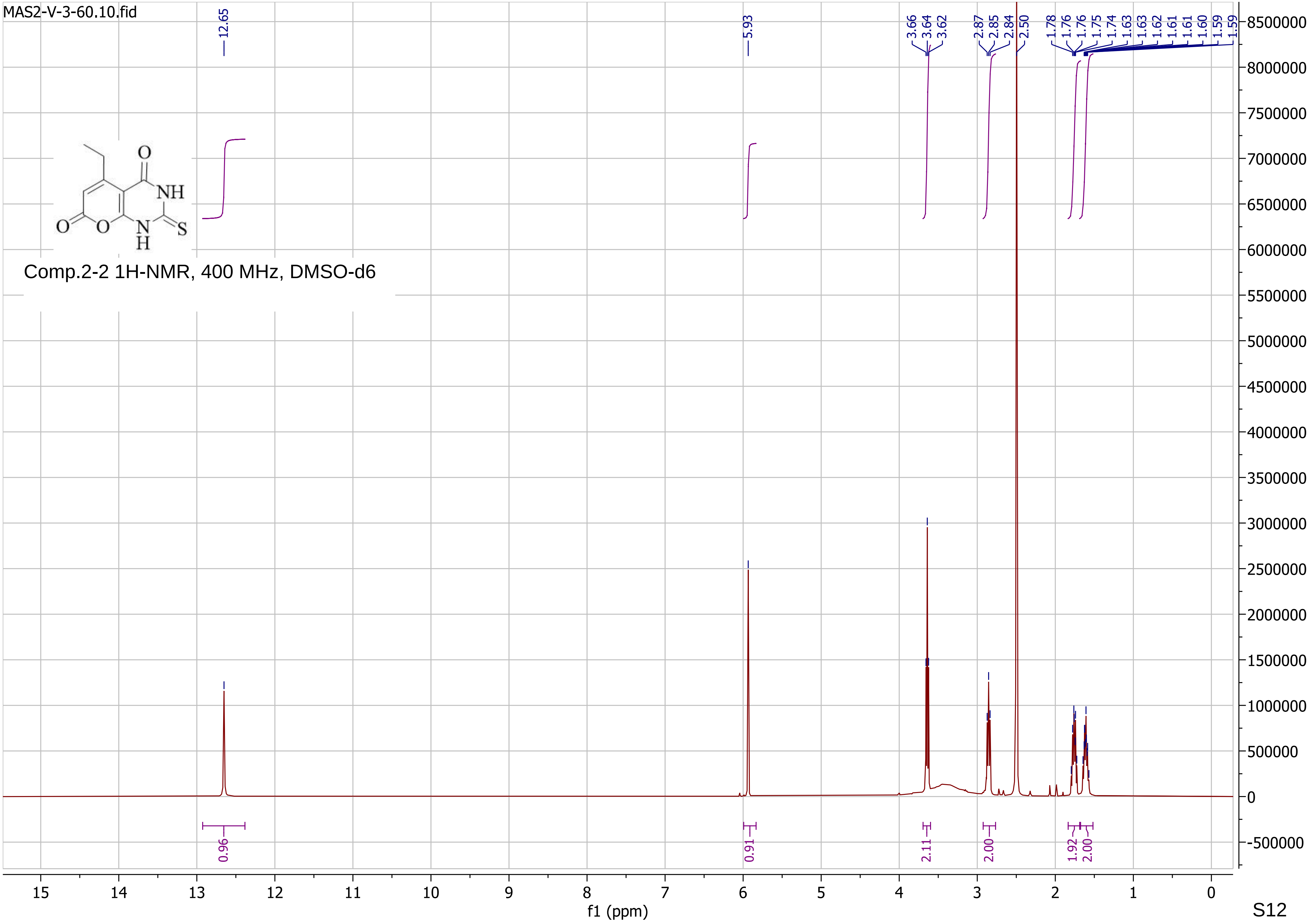

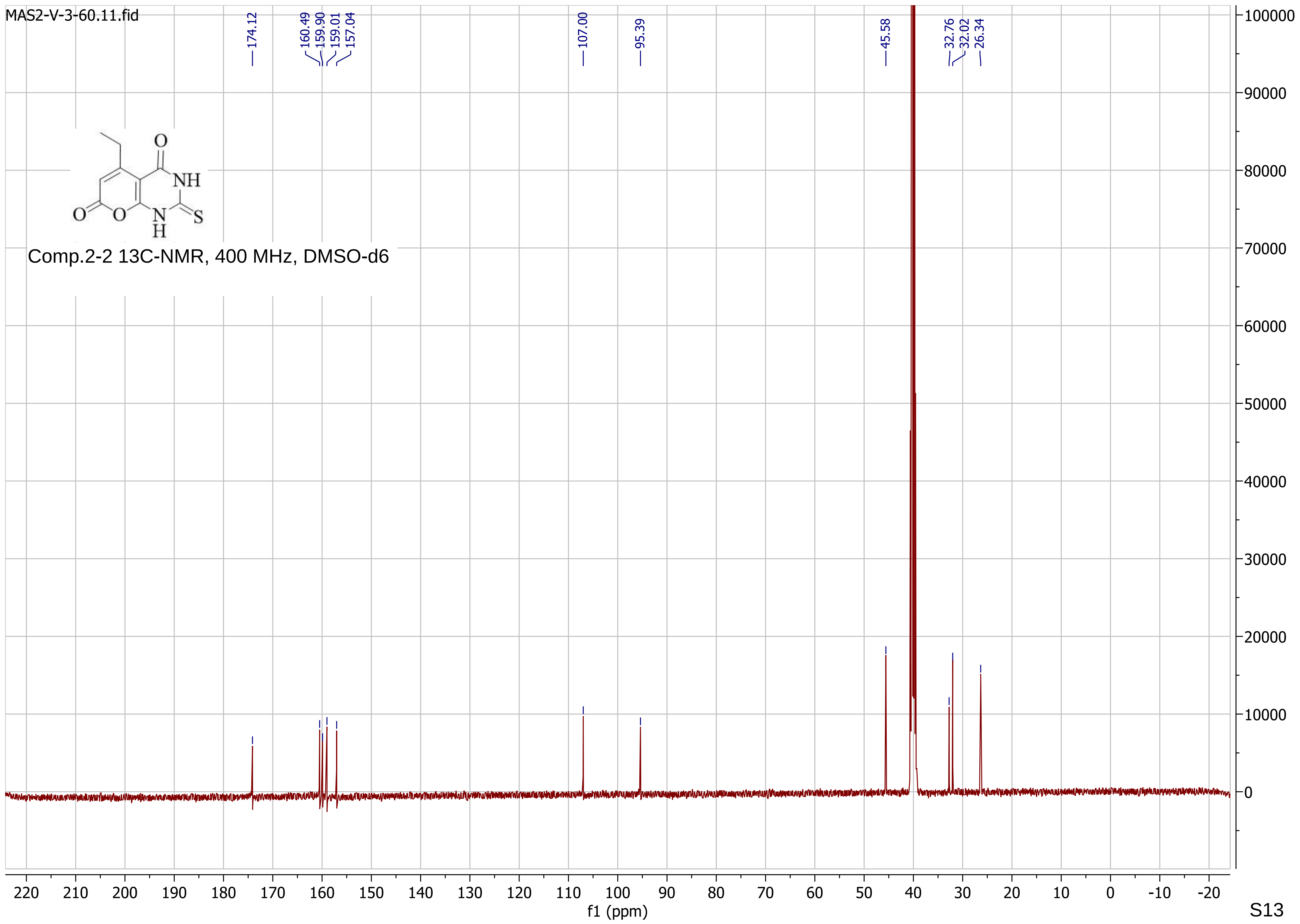
MAS2-V-3-60.11.fid
174.12
160.49
159.90
159.01
157.04
107.00
95.39
45.58
32.76
32.02
26.34
O
NH
S
N
H
O
O
Comp.2-2 13C-NMR, 400 MHz, DMSO-d6
f1 (ppm)

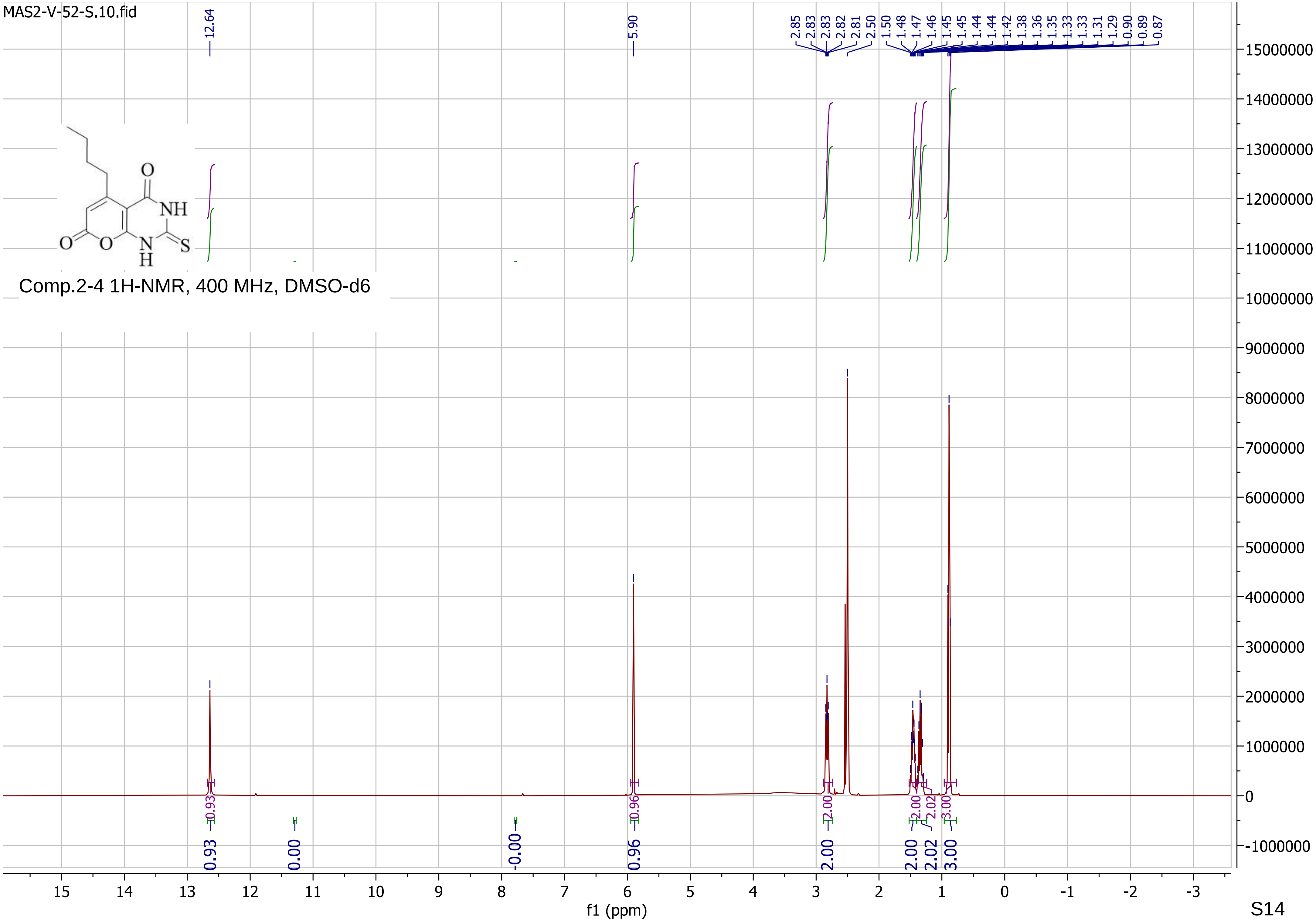

MAS2-V-52-S.10.fid
12.64
5.90
2.85
2.83
2.83
2.82
2.81
2.50
1.50
1.48
1.47
1.46
1.45
1.45
1.44
1.44
1.42
1.38
1.36
1.35
1.33
1.33
1.31
1.29
0.90
0.89
0.87
O
NH
O
O
N
H
S
Comp.2-4 1H-NMR, 400 MHz, DMSO-d6
0.93
0.00
-0.00
0.96
2.00
2.00
2.02
3.00
f1 (ppm)

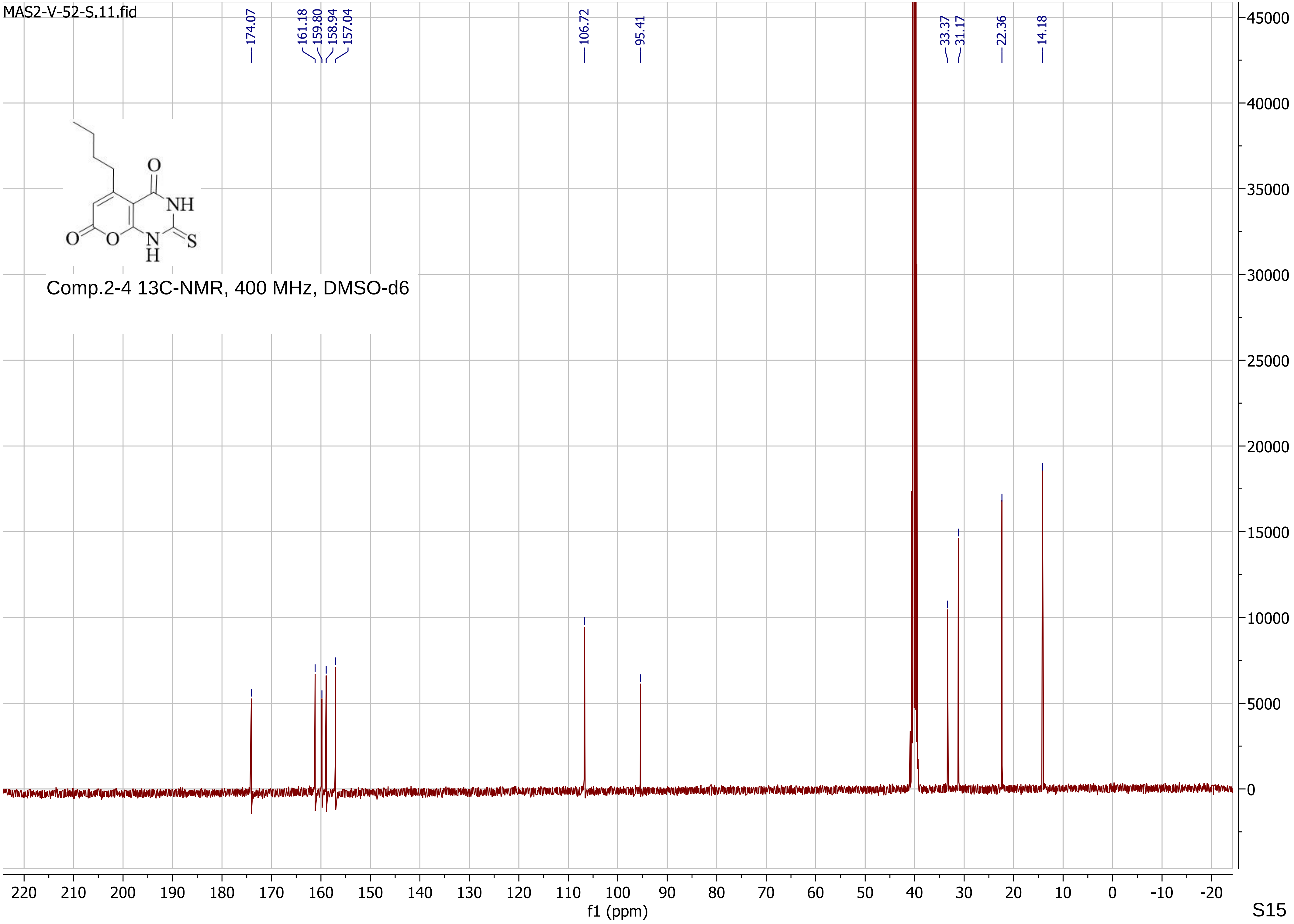

MAS2-V-52-S.11.fid
174.07
161.18
159.80
158.94
157.04
106.72
95.41
33.37
31.17
22.36
14.18
Comp.2-4 13C-NMR, 400 MHz, DMSO-d6
f1 (ppm)

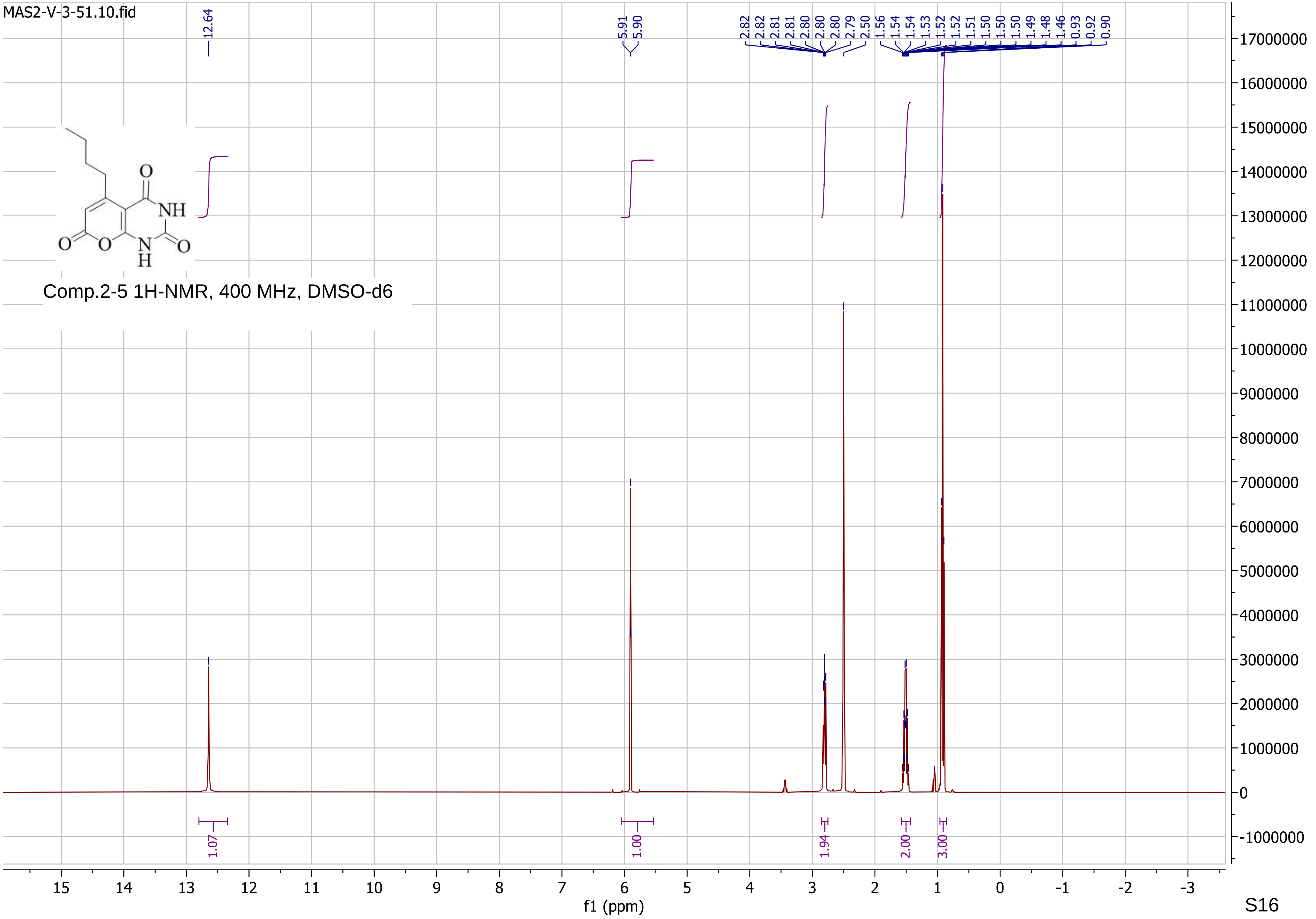
MAS2-V-3-51.10.fid
12.64
5.91
5.90
2.82
2.82
2.81
2.81
2.80
2.80
2.80
2.79
2.50
1.56
1.54
1.54
1.53
1.52
1.52
1.51
1.50
1.50
1.50
1.49
1.48
1.46
0.93
0.92
0.90
O
NH
O
O
N
H
O
Comp.2-5 1H-NMR, 400 MHz, DMSO-d6
1.07
1.00
1.94
2.00
3.00
f1 (ppm)

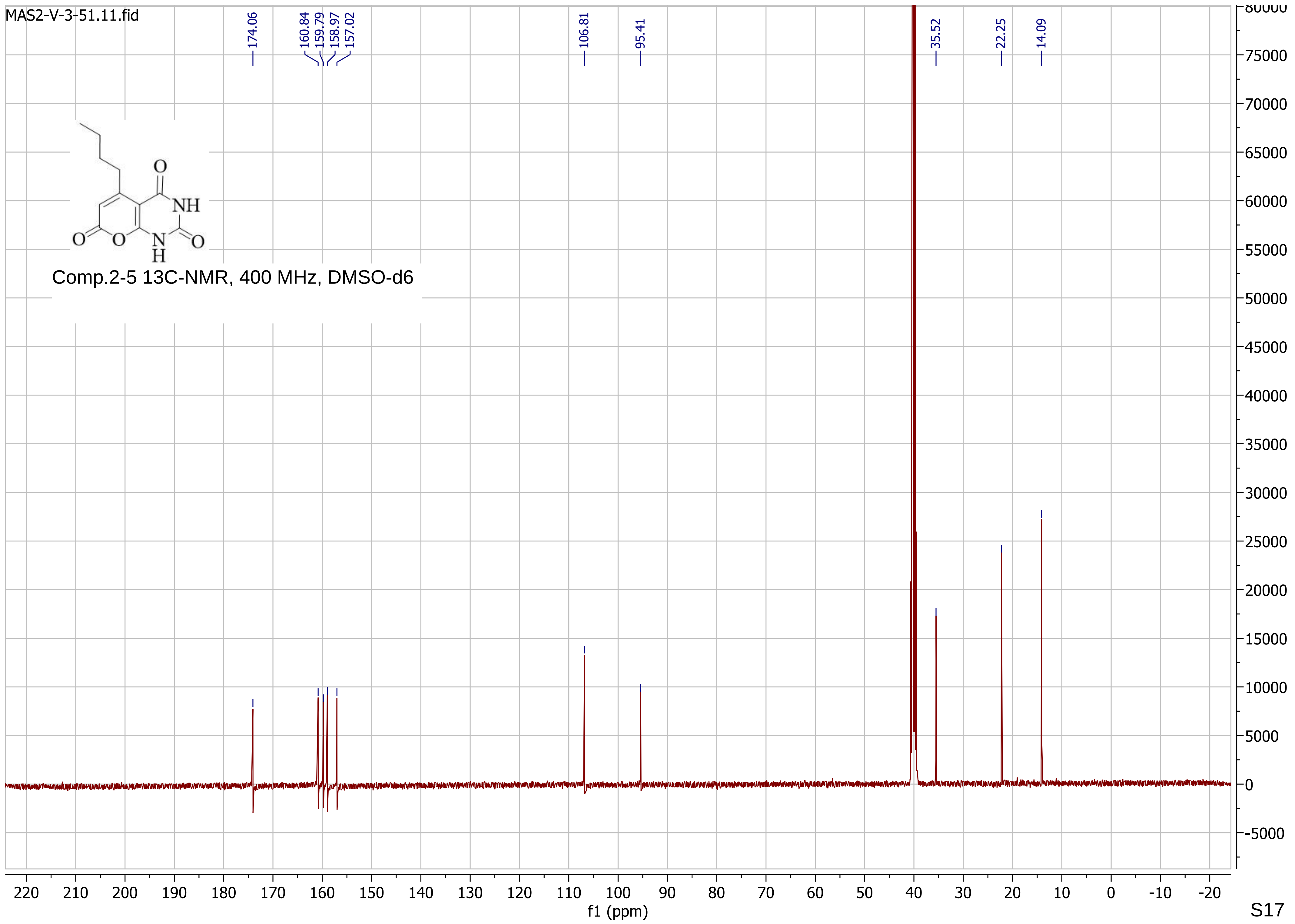
MAS2-V-3-51.11.fid
174.06
160.84
159.79
158.97
157.02
106.81
95.41
35.52
22.25
14.09
O
NH
O
O
N
H
O
Comp.2-5 13C-NMR, 400 MHz, DMSO-d6
220 210 200 190 180 170 160 150 140 130 120 110 100 90 80 70 60 50 40 30 20 10 0 -10 -20
f1 (ppm)
80000 75000 70000 65000 60000 55000 50000 45000 40000 35000 30000 25000 20000 15000 10000 5000 0 -5000

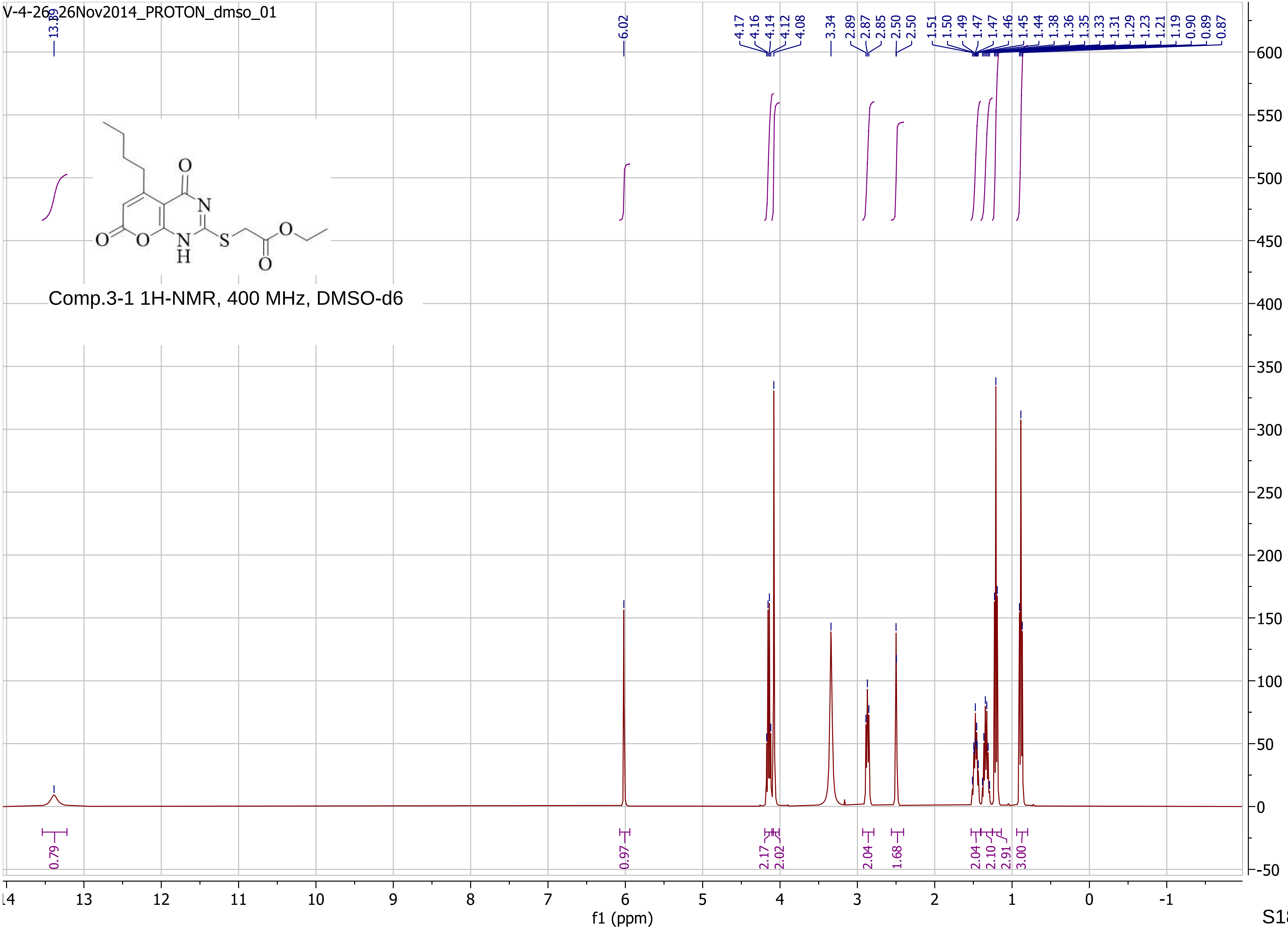
V-4-26_26Nov2014_PROTON_dmso_01
13.39
6.02
4.17
4.16
4.14
4.12
4.08
3.34
2.89
2.87
2.85
2.50
2.50
1.51
1.50
1.49
1.47
1.47
1.46
1.45
1.44
1.38
1.36
1.35
1.33
1.31
1.29
1.23
1.21
1.19
0.90
0.89
0.87
Comp.3-1 1H-NMR, 400 MHz, DMSO-d6
0.79
0.97
2.17
2.02
2.04
1.68
2.04
2.10
2.91
3.00
f1 (ppm)

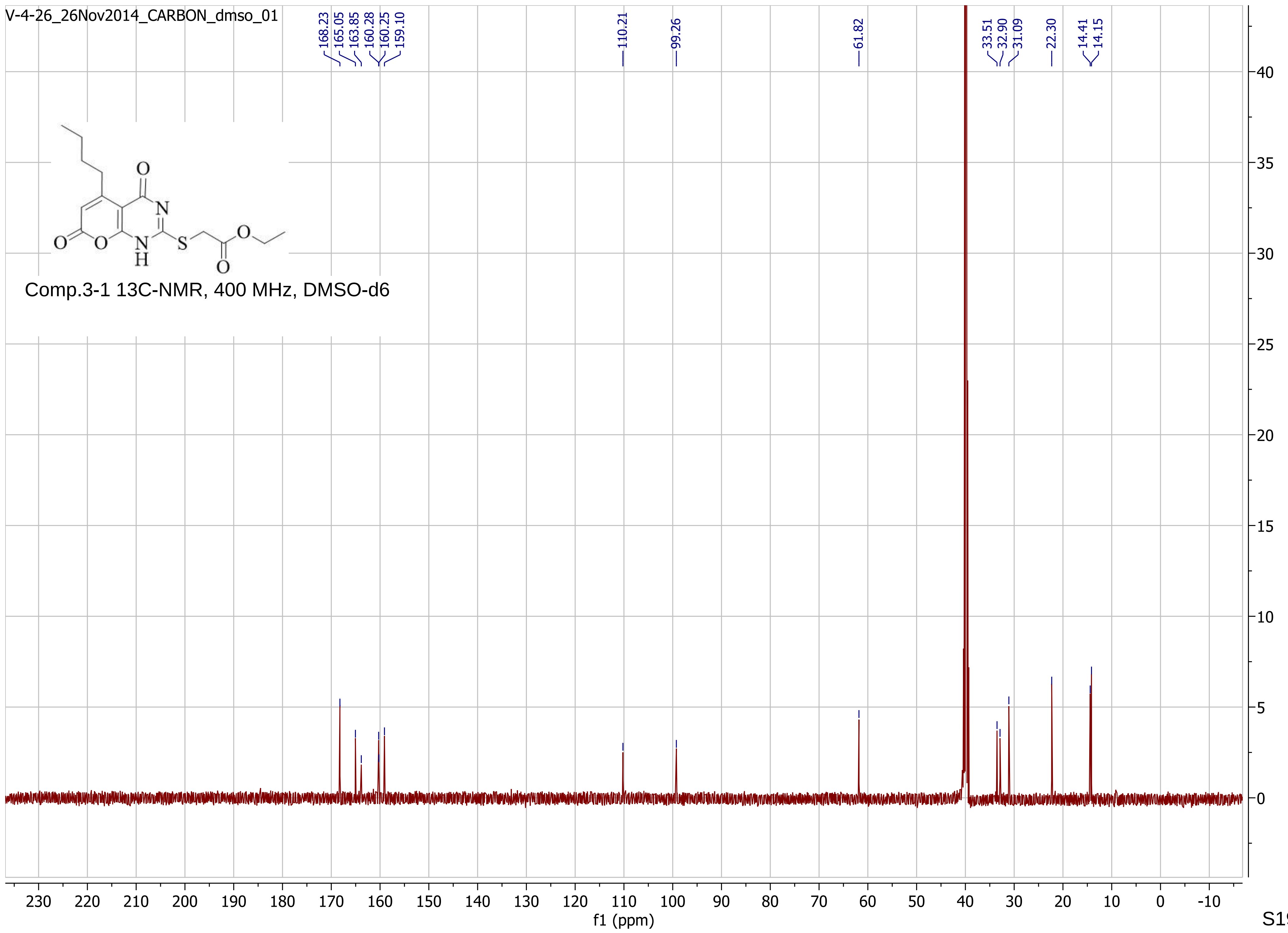
V-4-26_26Nov2014_CARBON_dmso_01
168.23
165.05
163.85
160.28
160.25
159.10
110.21
99.26
61.82
33.51
32.90
31.09
22.30
14.41
14.15
O
N
O
O
N
H
S
O
O
Comp.3-1 13C-NMR, 400 MHz, DMSO-d6
f1 (ppm)

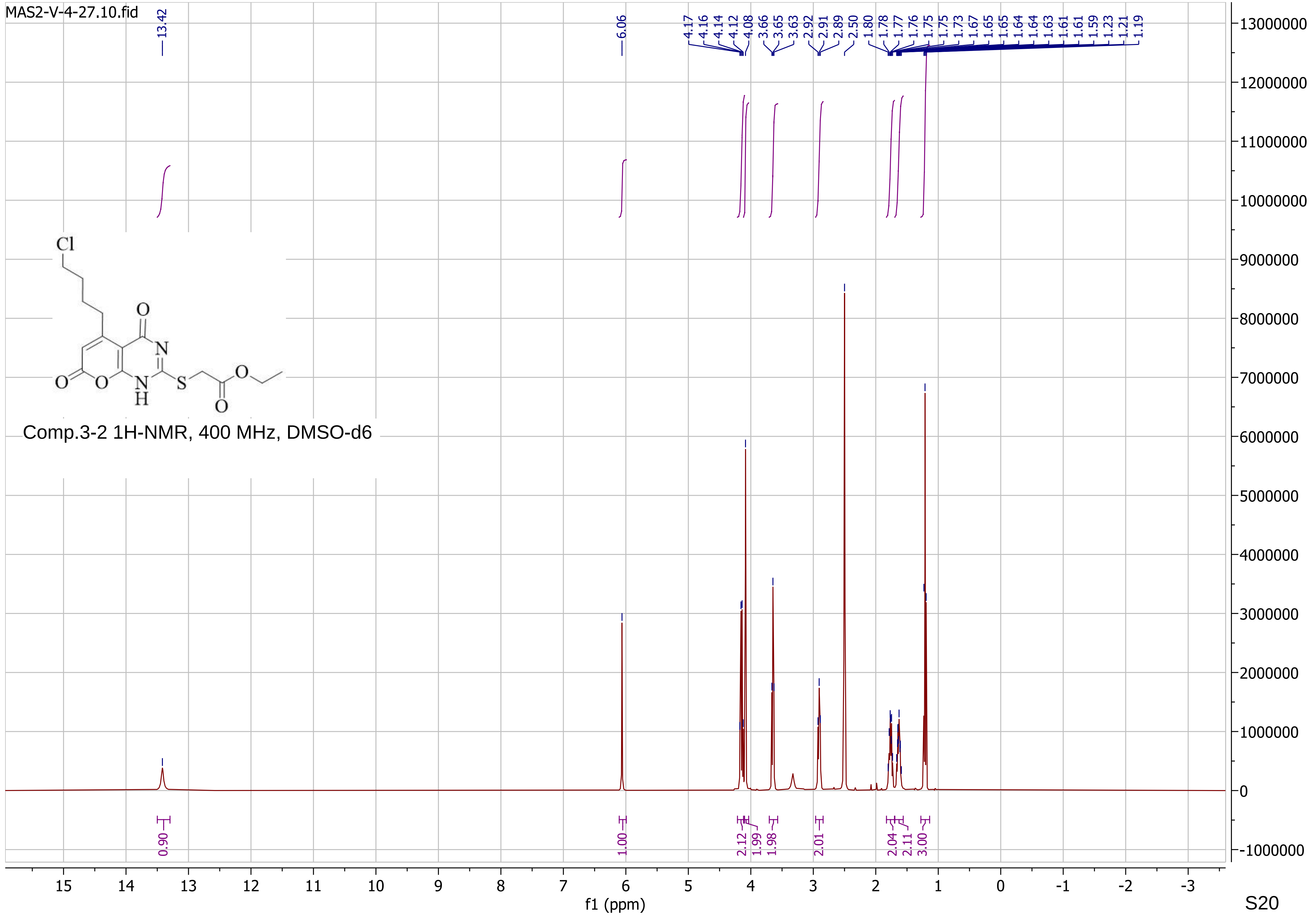
MAS2-V-4-27.10.fid
13.42
6.06
4.17 4.16 4.14 4.12 4.08 3.66 3.65 3.63 2.92 2.91 2.89 2.50 1.80 1.78 1.77 1.76 1.75 1.75 1.73 1.67 1.65 1.65 1.64 1.64 1.63 1.61 1.61 1.59 1.23 1.21 1.19
Cl
O
N
O
O
N
H
S
O
O
Comp.3-2 1H-NMR, 400 MHz, DMSO-d6
0.90
1.00
2.12
1.99
1.98
2.01
2.04
2.11
3.00
f1 (ppm)

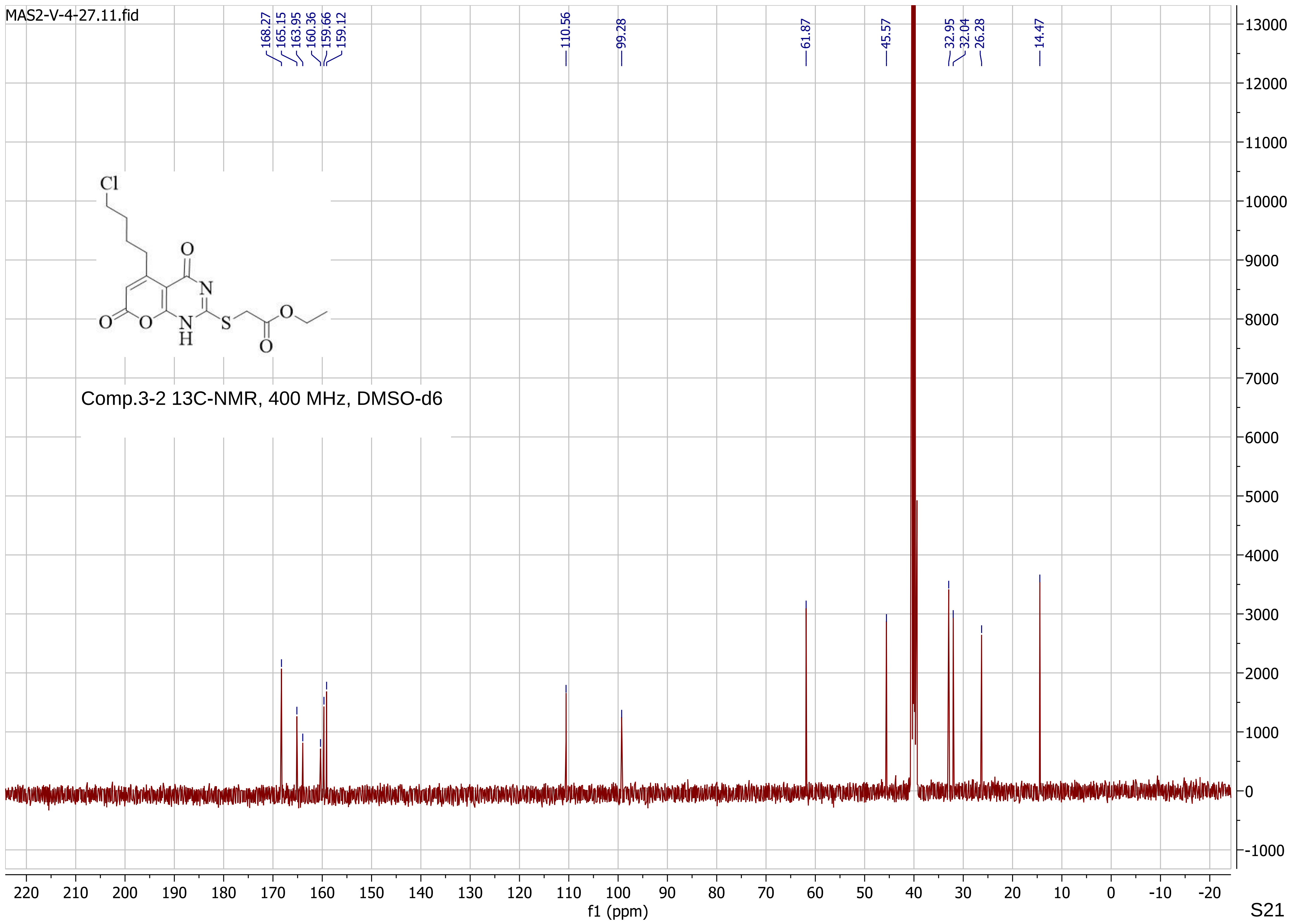
MAS2-V-4-27.11.fid
168.27
165.15
163.95
160.36
159.66
159.12
110.56
99.28
61.87
45.57
32.95
32.04
26.28
14.47
Cl
O
N
O
O
N
H
S
O
O
Comp.3-2 13C-NMR, 400 MHz, DMSO-d6
f1 (ppm)

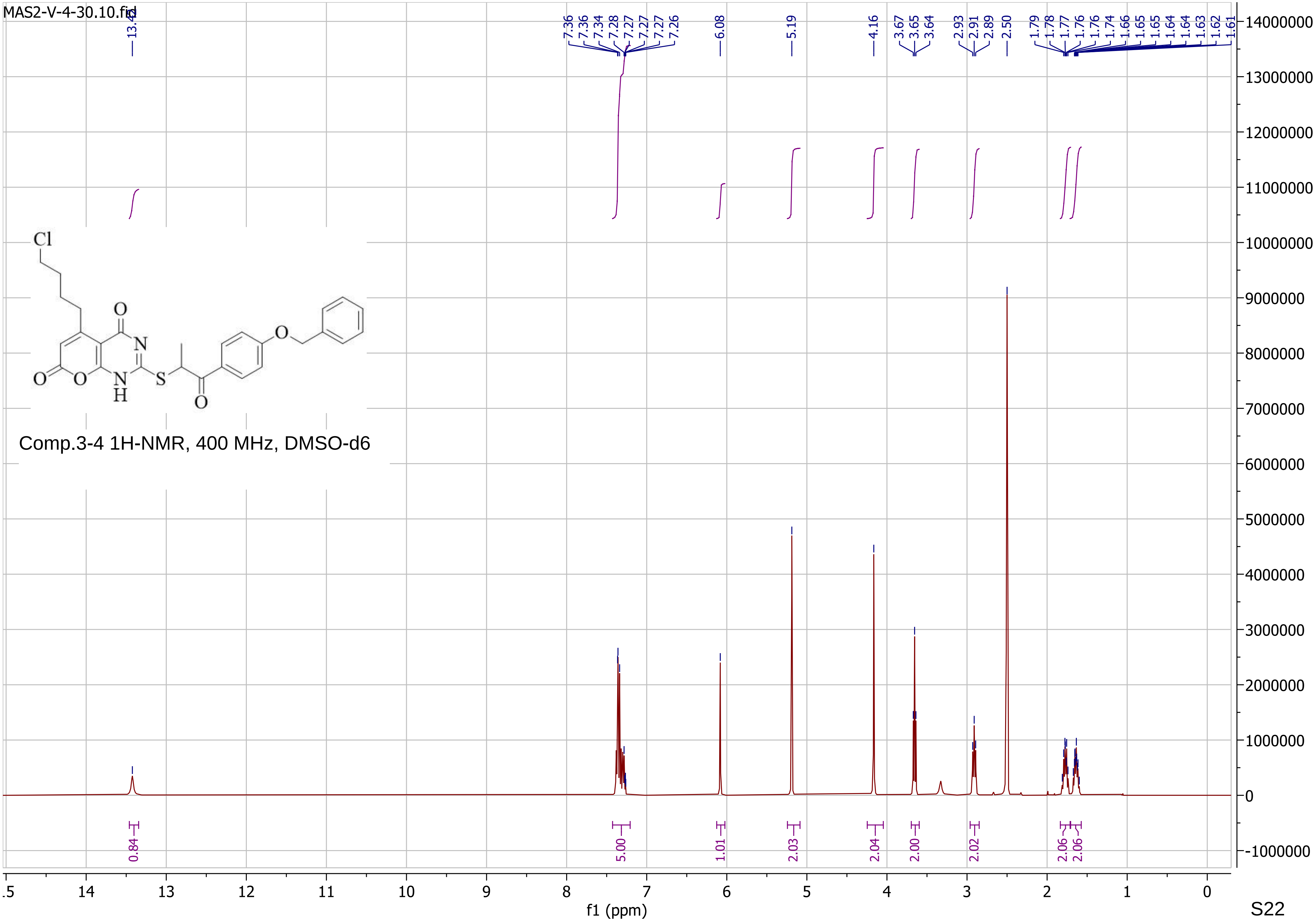

MAS2-V-4-30.10.fid
Comp.3-4 1H-NMR, 400 MHz, DMSO-d6
f1 (ppm)

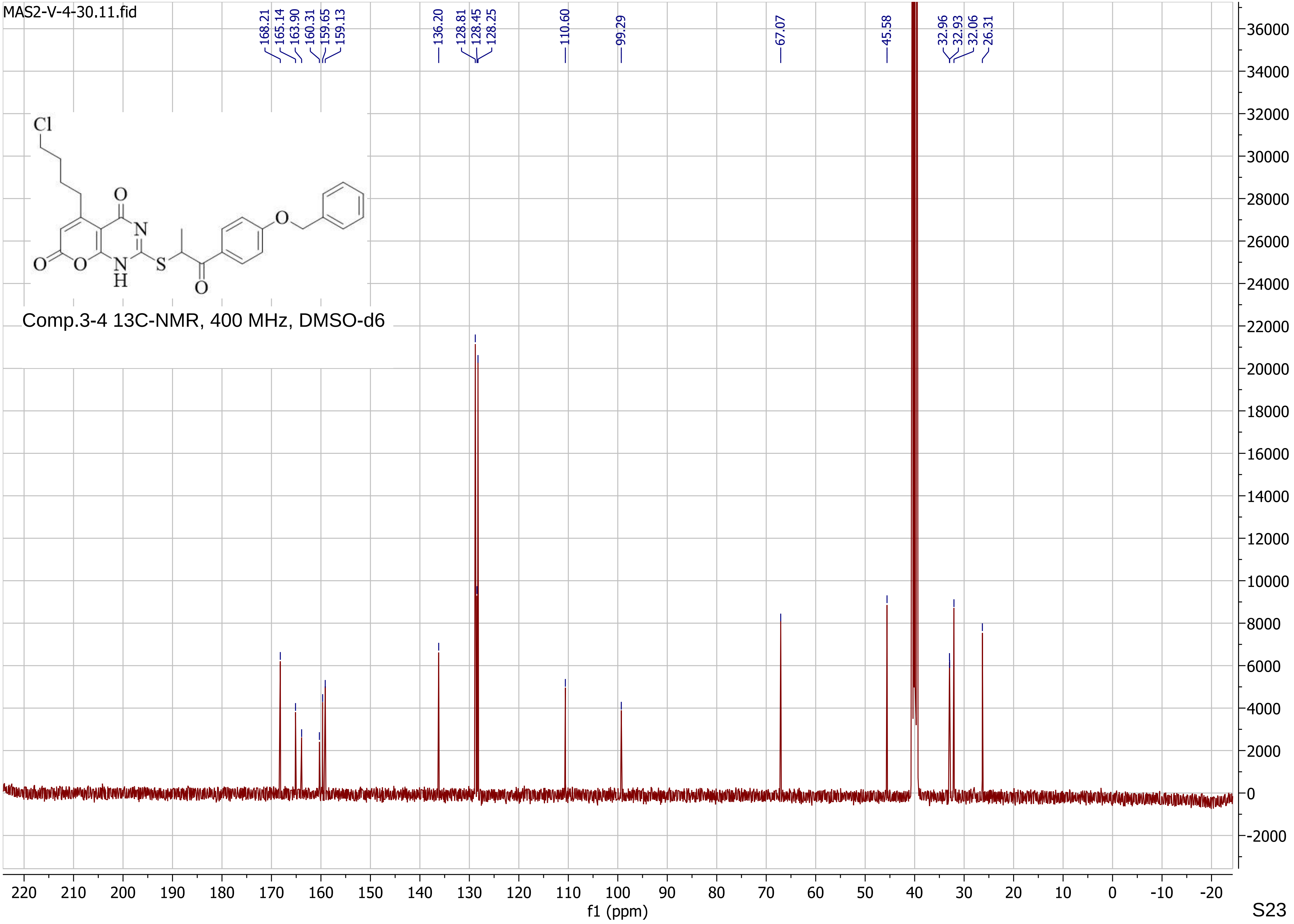
MAS2-V-4-30.11.fid
168.21
165.14
163.90
160.31
159.65
159.13
136.20
128.81
128.45
128.25
110.60
99.29
67.07
45.58
32.96
32.93
32.06
26.31
Cl
O
N
O
O
N
H
S
O
O
Comp.3-4 13C-NMR, 400 MHz, DMSO-d6
f1 (ppm)

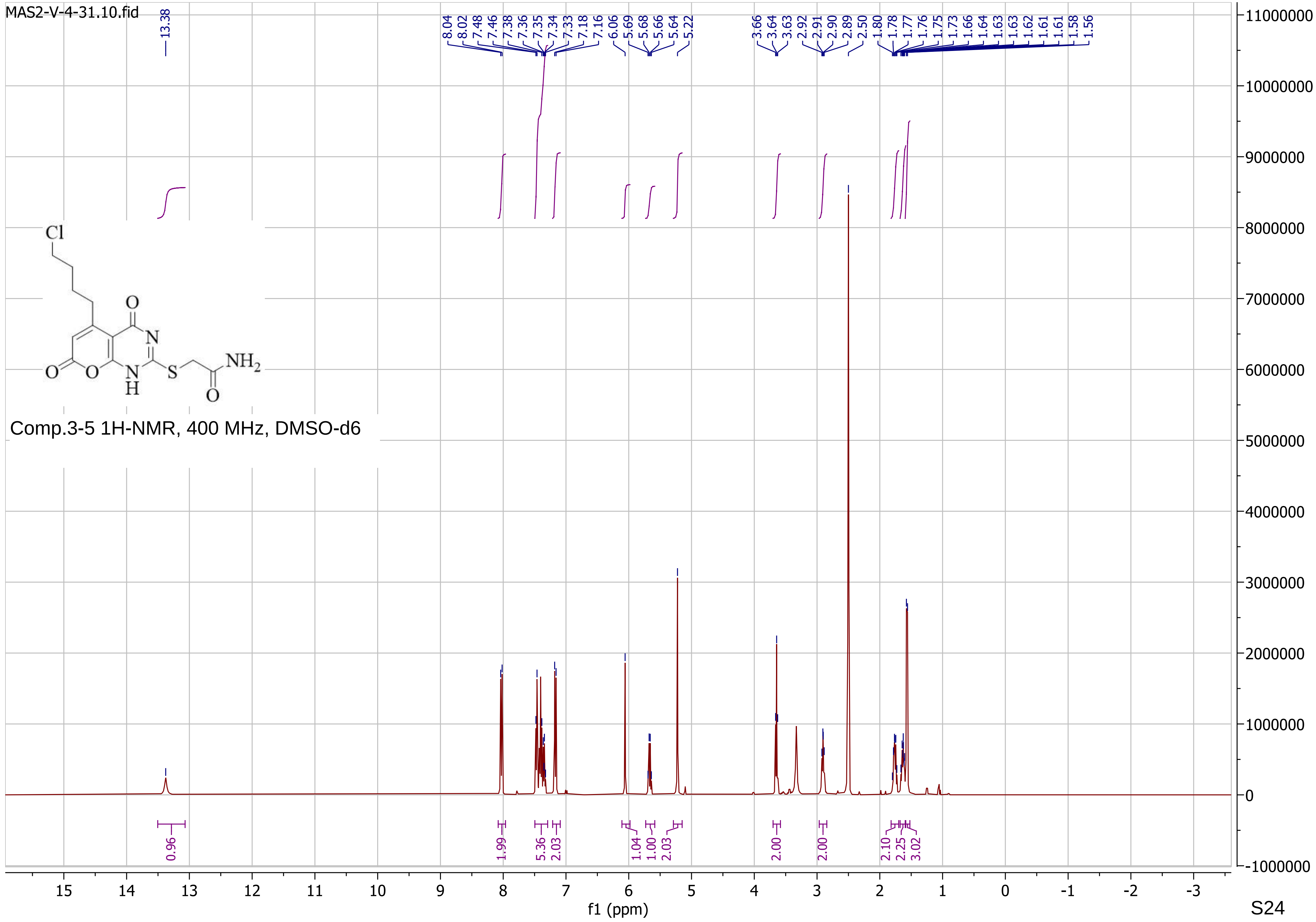
MAS2-V-4-31.10.fid
13.38
8.04 8.02 7.48 7.46 7.38 7.36 7.35 7.34 7.33 7.18 7.16 6.06 5.69 5.68 5.66 5.64 5.22
3.66 3.64 3.63 2.92 2.91 2.90 2.89 2.50 1.80 1.78 1.77 1.76 1.75 1.73 1.66 1.64 1.63 1.63 1.62 1.61 1.61 1.58 1.56
0.96 1.99 5.36 2.03 1.04 1.00 2.03 2.00 2.00 2.10 2.25 3.02
Cl
O
N
NH2
O
O
N
H
S
O
Comp.3-5 1H-NMR, 400 MHz, DMSO-d6
f1 (ppm)

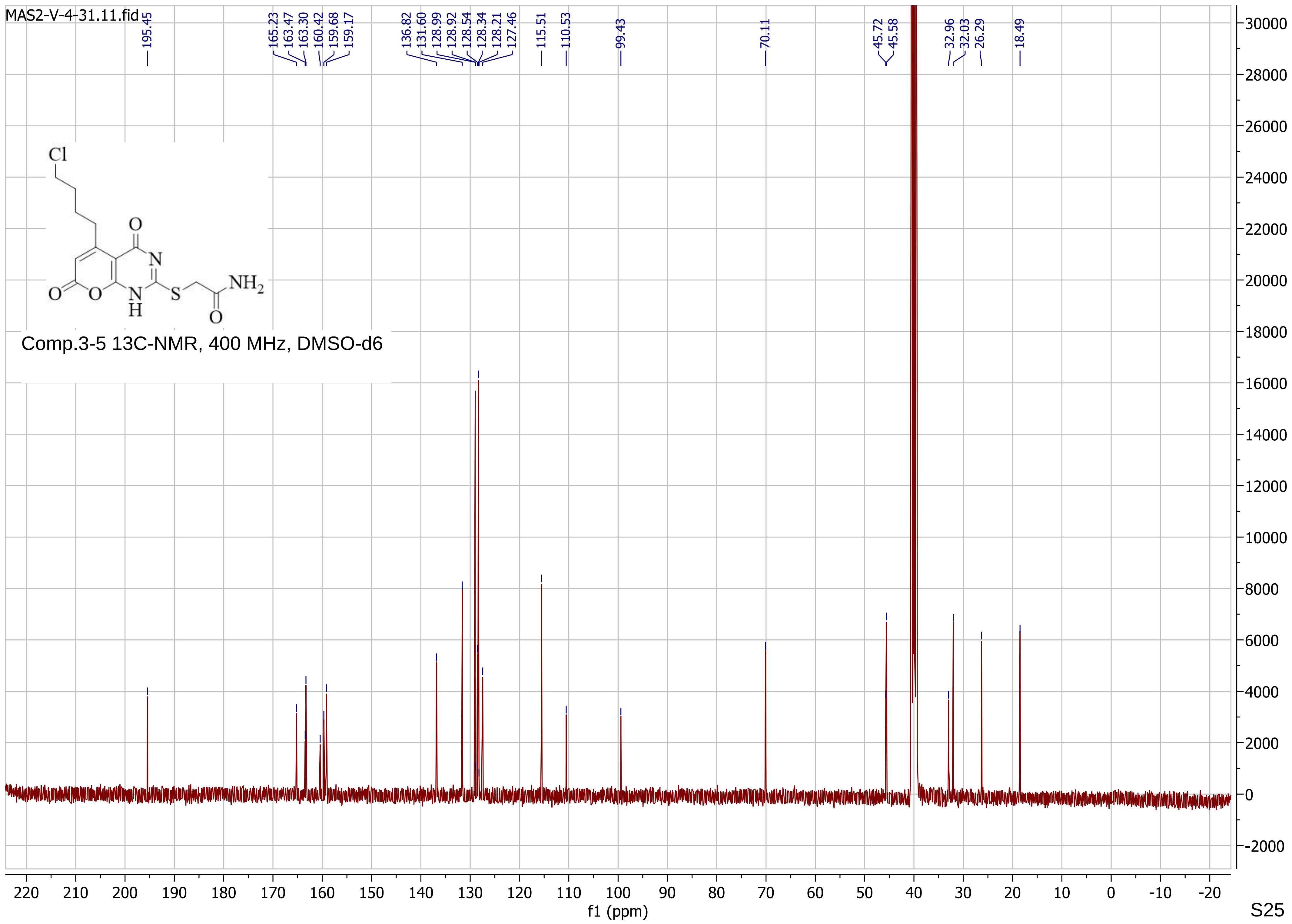
MAS2-V-4-31.11.fid
195.45
165.23
163.47
163.30
160.42
159.68
159.17
136.82
131.60
128.99
128.92
128.54
128.34
128.21
127.46
115.51
110.53
99.43
70.11
45.72
45.58
32.96
32.03
26.29
18.49
Cl
O
N
NH2
O
O
N
H
S
O
Comp.3-5 13C-NMR, 400 MHz, DMSO-d6
f1 (ppm)
